# Can polyploidy confer invasive plants with a wider climatic tolerance? A test using *Solidago canadensis*

**DOI:** 10.1002/ece3.6303

**Published:** 2020-05-26

**Authors:** Jizhong Wan, Ayub M. O. Oduor, Robin Pouteau, Beilei Wang, Luxi Chen, Beifen Yang, Feihai Yu, Junmin Li

**Affiliations:** ^1^ Zhejing Provincial Key Laboratory of Plant Evolutionary Ecology and Conservation Taizhou University Taizhou P.R. China; ^2^ State Key Laboratory of Plateau Ecology and Agriculture Qinghai University Xining P.R. China; ^3^ Department of Applied Biology Technical University of Kenya Nairobi Kenya

**Keywords:** climatic envelope, invasive plant, plant functional trait variation, polyploidy

## Abstract

Polyploidy can cause variation in plant functional traits and thereby generate individuals that can adapt to fluctuating environments and exploit new environments. However, few empirical studies have tested for an association between ploidy level and climatic tolerance of invasive cytotypes relative to conspecific native‐range cytotypes. Here, we used an invasive plant *Solidago canadensis* to test whether invasive populations had a higher proportion of polyploids, greater height and stem‐base diameter, and occupied a wider range of climatic conditions than conspecific native‐range populations. We also tested whether the invasive populations had overcome genetic founder effects. We sampled a total of 80 populations in parts of the invaded range in China and native range in North America for in situ measurements of plant height and stem‐base diameter in the field and for population genetic and cytotype analyses. To examine climatic correlates, we augmented our field‐sampled data with occurrence records obtained from Global Biodiversity Information Facility. All, except one, of the populations that we sampled in China occurred in a humid subtropical climate. In contrast, the North American populations occurred in humid continental, humid subtropical, and semi‐arid climatic zones. All populations of *S. canadensis* in China were purely hexaploid, while the North American populations were diploid, tetraploid, and hexaploid. The invasive hexaploids were significantly taller and had a larger stem‐base diameter than native hexaploids. Native hexaploids were significantly taller and had larger stem‐base diameter than native diploids. Climatic correlate assessment found that invasive and native populations occupied different climatic envelopes, with invasive populations occurring in warmer and less seasonal climates than native populations. However, there was no significant correlation between ploidy level and climatic envelope of *S. canadensis*. Molecular phylogeography data suggest reduced genetic founder effects in the invaded range. Overall, these results suggest that polyploidy does not influence *S. canadensis* climatic tolerance.

## Graphical abstract

Climatic envelope assessment found that invasive and native populations occupied different climatic niches, with invasive populations preferring warmer and less seasonal climates than native populations. Molecular phylogeography data provide signatures of reduced genetic founder effects in the invaded range. Polyploidy does not influence *Solidago canadensis* climatic niche widths.

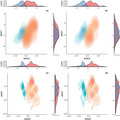

## 1 INTRODUCTION

Invasions by exotic plant species pose substantial threats to native biodiversity and alter ecosystem processes across a broad range of environments (McGeoch et al., 2010; Pyšek et al., 2012). To improve effectiveness of invasive plant management, it is necessary to understand the mechanisms that underpin invasive spread of exotic plants (Dawson, Burslem, & Hulme, 2009). Species with high variation in functional traits (morphological, physiological, and phenological) can adapt to different stress levels and increase the uptake of limiting resources across a broad range of climatic conditions (Nicotra et al., 2010). Thus, plants that possess higher mean values of, and variation in, functional traits in the invaded ranges than in their native ranges may have an enhanced capacity to colonize a wide range of climatic conditions (Pyšek et al., 2012; Richardson & Pyšek, 2006). Therefore, an understanding of the factors that determine variation in functional trait values in invasive plants is key to predicting invasion risks (Leffler, James, Monaco, & Sheley, 2014; te Beest, Esler, & Richardson, 2015).

Polyploidy (i.e., whole genome duplication) can cause variation in plant morphology, phenology, and physiology and thereby generate individuals that can adapt to fluctuating environments, exploit new environments, or outcompete progenitor diploids (te Beest et al., 2012; Simón‐Porcar, Silva, Meeus, Higgins, & Vallejo‐Marín, 2017; Soltis, Visger, & Soltis, 2014). Therefore, polyploidy may confer plants with novel features that allow them to invade new environments or expand their ecological ranges (Parisod, Holderegger, & Brochmann, 2010; Thébault, Gillet, Müller‐Schärer, & Buttler, 2011; te Beest et al., 2015). Several studies have found invasive plant species to have higher ploidy levels in the introduced ranges than in their native ranges. For instance, this has been reported for invasions by *Centaurea maculosa* (Muller, 1989; Treier et al., 2009), *Lythrum salicaria* (Kubatova et al., 2008), and *Centaurea stoebe* in North America (Mráz, Tarbush, & Müller‐Schärer, 2014); *Solidago gigantea* (Schlaepfer, Edwards, & Billeter, 2010) and *Senecio inaequidens* in Europe (Monty, Maurice, & Mahy, 2010); *Solidago canadensis* in China (Li, Liu, Yan, & Du, 2017); and *Lilium lancifolium* in Europe and North America (Chung et al., 2015). However, few empirical studies have tested for an association between variation in ploidy level and climatic tolerance of invasive plants in their introduced and native ranges (Broennimann, Mráz, Petitpierre, Guisan, & Müller‐Schärer, 2014; Treier et al., 2009). A case study on *C. maculosa* found that invasive‐range tetraploid populations occupied a drier climate than conspecific native‐range tetraploid populations (Treier et al., 2009). Nevertheless, later experimental results suggested that drought tolerance did not differ between *C. maculosa* cytotypes, but was instead correlated with original latitudinal clines within cytotypes (Mráz et al., 2014). The paucity of studies that integrate cytobiogeography with evaluation of climatic tolerance pre‐empts drawing a firm conclusion as to whether higher ploidy levels confer wider climatic tolerance.

It also remains unresolved whether a pronounced shift in cytotype distribution that has been reported in several invasive plant species is due to stochastic genetic founder events or adaptive evolutionary processes. Population genetic theory predicts that invasive species are likely to experience genetic founder effects, which may reduce their ability to adapt to novel environmental conditions (Dlugosch & Parker, 2008). Founder effects can cause a reduction in genetic diversity because the introduced populations are often a subset of the original, larger populations (Dlugosch & Parker, 2008). Nevertheless, studies have found evidence for local adaptation in several invasive species (Oduor, Leimu, & Kleunen, 2016), which implies that the species have overcome genetic founder effects. Invasive species may overcome (or at least minimize) founder effects through multiple introduction events (i.e., introductions of diverse genetic lineages from different source populations in the native range) or a single introduction event from a native‐range source population having high genetic variation (Genton, Shykoff, & Giraud, 2005; Le Roux et al., 2011; Meyerson & Cronin, 2013; Oduor et al., 2015). However, few studies have used cytobiogeographical and molecular phylogeographical approaches to determine whether invasive cytotypes represent a full complement of genetic diversity that occurs in the native range.

Here, we used the invasive plant *S. canadensis* to test a prediction that polyploidy can enable invasive plant species to colonize a wider range of climatic conditions. Specifically, we combined a cytobiogeographical approach, estimates of functional traits and climatic correlates, and molecular phylogeography methods to test whether invasive populations of *S. canadensis:* 1) had a higher proportion of polyploids, 2) were larger, 3) had a wider climatic tolerance, and 4) had overcome founder effects, relative to native populations of *S. canadensis*.

## 2 MATERIALS AND METHODS

### 2.1 Study species

*Solidago canadensis* L. (Asteraceae) is a rhizomatous clonal perennial forb native to North America and has invaded China and other regions (Europe, Australia, New Zealand, and Japan) (Abhilasha, Quintana, Vivanco, & Joshi, 2008; Jin, Yuan, Gao, Oduor, & Li, 2020; Xu & Qiang, 2011). *Solidago canadensis* reproduces both clonally and sexually (Li et al., 2017). The plant is self‐incompatible and produces small wind‐dispersed seeds for long‐distance dispersal (Li et al., 2017). However, the plant can spread clonally at a local scale (Li et al., 2017). In China, *S. canadensis* was first introduced to Zhejiang, Jiangsu, and Shanghai provinces in the early 1930s as an ornamental garden species (Xu et al., 2012; Zhao et al., 2015). It escaped from cultivation and experienced explosive spread across eastern China after 1980s, and is now distributed widely across eastern China where it abounds in disturbed habitats, including agricultural lands, roadsides, railways, and city suburbs (Dong, Yu, & He, 2015; Zhao et al., 2015). The species has diploid, tetraploid, and hexaploid cytotypes in the native range, but only hexaploids have been found in China (Li et al., 2017). Previous studies (Xu et al., 2012; Zhao et al., 2015) suggest that the Chinese populations were founded through multiple introduction events.

### 2.2 Sampling and functional trait measurement

To identify occurrence locations of *S. canadensis* in China and North America, we conducted field surveys in summer and autumn for the period 2013–2016. We sampled a total of 2,051 *S. canadensis* individuals in 19 native and 61 invasive populations. The number of individuals sampled per population ranged from five to 33. The native range in North America was represented by 577 individuals, while the invaded range in China was represented by 1,474 individuals (Appendix 1). Any two sample populations were at least 5 km apart. In each population, we sampled at random plants that occurred within a radius of 10–20 m to minimize a potential bias due to environmental heterogeneity within a population (e.g., soil moisture and nutrients). To avoid sampling plants from the same genet within the same radius, we sampled plants that were at least two meters apart from each other. For each *S*. *canadensis* individual, we took in situ measurements of stem‐base diameter and plant height (in cm). These functional traits are generally linked to ecological strategy axes of plants. As a plant developmental stage (i.e., phenophase) can influence the values of these traits, we took measurements only from mature plants that had set seeds and were therefore presumably not undergoing further development in height and stem diameter. We omitted leaf measurements because an individual *S. canadensis* plant can have hundreds of leaves (especially in the non‐native range), and hence, finding the largest leaf for measurement can be extremely difficult. In the native range, the ecological niches of *S. canadensis* and those of its congeners *S. gigantea* and *S. altissima* overlap and the three species can coexist (Benelli et al., 2019; Weber, 1998). However, *S. canadensis* prefers loose and drier soils than the congeners, and hence, it occurs mostly near urban areas, roadsides, and railways, while the congeners occur mainly on riverside and embankments (Benelli et al., 2019). Therefore, within a locality, we sampled only the preferred habitats of *S. canadensis* to avoid accidental sampling of the congeners. Moreover, we conducted a test of phylogenetic relatedness among the populations that we sampled in the field to determine whether they all belonged to *S. canadensis* (see the section on population genetics and phylogenetic analyses below). All the sampled populations in China occurred in a humid subtropical climate except one that occurred in a subtropical highland climate. In contrast, the North American populations were sampled from humid continental, humid subtropical, and semi‐arid climatic zones (Köppen–Geiger climate classification system) (Kottek, Grieser, Beck, Rudolf, & Rube, 2006).

### 2.3 Determination of ploidy level

We took fresh leaf samples from the same *S. canadensis* individuals used for functional trait measurements described above. The leaf materials were held in self‐sealing plastic bags containing silica and transported to the laboratory for ploidy level determination. We applied a modified flow cytometry method by Suda and Trávníček (2006) in the determination of ploidy level. In brief, 20 mg of silica‐dried leaf tissue was chopped up with a razor blade in 1 ml of ice‐cold extraction buffer (20 mM MOPS, 45 mM MgCl_2_, 30 mM sodium citrate, 0.1% v/v Triton X‐100, 0.5% w/v polyvinylpyrrolidone) in a plastic Petri dish held on a chilled brick. A new razor blade was used for each leaf sample. The resultant fine slurry was filtered through a 600‐mesh nylon filter (Shanghai Aoran Hardware Market, Shanghai, China). The filtrate was held in a new 5‐ml centrifuge tube. The filtered slurry was then resuspended in a pre‐cooled 1 ml of extraction buffer in a Petri dish and then filtered again. The final filtrate was treated with 10 μL/ml of RNase A for 10 min at 4°C. Then, 200 μL/ml of a propidium iodide (PI) staining solution was added and incubated at 4°C for 30 min in darkness. The fluorescence strength of PI‐DNA conjugates was measured on Attune® NxT Acoustic Focusing Flow Cytometer (Thermo Fisher Scientific Inc. Waltham, MA, USA) using a 488 nm laser and 590/40 emission filter. Leaf of the hexaploid *S. canadensis* (confirmed by cytometry and karyotype analysis) collected in Taizhou City, Zhejiang Province, China, was used as an external reference.

### 2.4 Statistical analyses

To test whether *S. canadensis* plants from the invaded range had significantly greater height and stem‐base diameter than *S. canadensis* plants from the native range, we fitted linear mixed‐effect models. In the models, a trait value was treated as a dependent variable, while *S. canadensis* range (invaded vs. native) was treated as a fixed‐effect independent variable. Population identity of *S. canadensis* was treated as a random‐effect factor and nested within range. To test whether polyploidy in general conferred higher mean trait values, we also fitted linear mixed‐effect models to test whether the functional trait values differed between hexaploid and diploid individuals in the native range. Because only hexaploids were found in the invaded range, this latter test was not possible for invasive plants. In the test, ploidy level (polyploid vs. diploid) was treated as a fixed‐effect independent variable, while *S. canadensis* population identity was treated as a random‐effect independent variable. We did additional analyses to test for the potential confounding effect of climate on trait expression by comparing traits of invasive plants with those of native plants from the same climatic zone only. As nearly all invasive plants were sampled in a humid subtropical climate, we used a subset of plants from this climatic zone only. We did separate comparisons for groups of plants that occurred in populations with mixed‐ploidy and for groups of plants that occurred in populations with one ploidy type. Moreover, we compared traits of diploid plants with those of hexaploid plants that occurred in mixed‐ploidy populations only to exclude the potential confounding effect of pure and mixed‐ploidy populations occupying habitats with contrasting ecological conditions. In all the analyses, we fitted the models using maximum likelihood with the *lme* function in the *nlme* package (Pinheiro, Bates, DebRoy, & Sarkar, 2007) in r v 3.5.2 (R Core Team, 2018). We used likelihood ratio tests to assess the significance of each factor by comparing a model without the factor with a full model.

### 2.5 Assessing climatic tolerance

We described the climatic space occupied by *S. canadensis* with 19 variables with a 30 arc‐sec resolution (~1 km) derived from the WorldClim database (Hijmans, Cameron, Parra, Jones, & Jarvis, 2005). After standardization of the climatic variables (mean equal to zero and standard deviation equal to 1), we reduced the dimensionality of the climatic hyperspace to two axes (PC1 and PC2) based on a PCA (Broennimann et al., 2012). In addition to our 80 field‐sampled records of *S. canadensis* in China and North America, we collected information on occurrences of *S. canadensis* provided by the Global Biodiversity Information Facility (GBIF) database (http://www.gbif.org/) in order to assess more comprehensively the climatic tolerance of the species. We retained only the GBIF occurrence records in the native (United States, Canada, and Mexico) and invaded (Australia, New Zealand, Japan, and China) ranges where the species is most widespread. Occurrences flagged as invalid (e.g., in the oceans) or with doubtful coordinates were removed as well as those separated by less than 30 arc‐seconds to avoid pseudo‐replication. We then extracted values of PC1 and PC2 for each of the 2,214 retained occurrence locations (i.e., our 80 field‐sampled records and 2,134 GBIF records, which included 1,624 records in the native range and 510 records in the invaded range).

To test whether climatic space occupied by *S. canadensis* differed significantly between the invaded and native ranges, we calculated the kernel‐smoothed density of occurrence of the two ranges based on the density of the different combinations of climatic conditions available to each range (Broennimann et al., 2012). The overlap between the climatic envelope of the two ranges was estimated with the Schoener's *D* metric, which ranges from 0 (no envelope overlap) to 1 (identical envelopes) (Schoener, 1968). We then tested for envelope equivalency and the less restrictive hypothesis of envelope similarity (comparing the observed values of envelope overlap to the 95th percentile density of the simulated values) (Warren, Glor, & Turelli, 2008). To achieve this, the *D* value between the two ranges was compared to a null distribution of *D* values computed between simulated envelopes built through randomization procedures (10,000 randomly selected “background” points generated 100 times). This was computed with the r package “ecospat” (Di Cola et al., 2017). As a *D* value does not allow a determination of whether an absence of envelope overlap results from the lack of overlap for one or two PCA axes, we used the function “niceOverPlot” implemented in R for easier interpretation. To test whether *S. canadensis* inhabited a wider range of climatic conditions in its invaded range than in the native range, we performed an *F‐*test of equality of variances (homoscedasticity) along the two PCA axes. We also used the same method to compare the climatic zones occupied by diploid and hexaploid populations of *S. canadensis* in its native range. Finally, we compared *D* values and envelope equivalencies and similarities between the following groups of *S. canadensis* populations: 1) North American versus Chinese populations that we had sampled in the field and with the same ploidy level, 2) North American versus Chinese populations that we had sampled in the field and without controlling for variation in ploidy level, 3) a global data set (i.e., the 80 populations that we had sampled in the field and GBIF records) of North America and Mexico grouped together versus China, and 4) a global data set of North America and Mexico grouped together versus all occurrence records in Australia, New Zealand, Japan, and China. Comparisons 1 and 2 tested the potential effect of variation in ploidy, while comparisons 3 and 4 tested whether our sampling was representative of the invaded and native ranges. For comparisons 3 and 4, it was not possible to control for variation in ploidy level because information on ploidy level was missing in the GBIF database.

### 2.6 Population genetic and phylogenetic analyses

#### 2.6.1 DNA extraction, PCR, and sequencing

To estimate gene flow in *S. canadensis* from North America to China and phylogenetic relationships among the 80 invasive and native populations that we sampled in the field, we sequenced two chloroplast spacer regions (*psbA‐trnH* and *trnL‐F*) using four universal primer pairs (Appendix 2) (de Andrade et al., 2010). We sequenced 8–14 individuals per population. The primers were synthesized by Sangon Biotechnology Company (Shanghai, China). The PCR mix with a total volume of 20 μl comprised: 1 × PCR buffer, 2.5 mM Mg^2+^, 4 ng template DNA, 0.15 μM each for forward and reverse primer, 0.3 mM 4 × dNTP mixture, and 1.5 U Taq polymerase (Promega Cooperation, Madison, Wisconsin, USA). PCR amplifications were performed in a PTC 220 Thermal Cycler (Bio‐Rad Laboratories, Hercules, California, USA) as follows: denaturation at 95°C for 5 min, followed by 40 cycles of 45 s at 94°C, 45 s at 57°C, 90 s at 72°C, with a final elongation of 20 min at 72°C. PCR products were then sequenced using ABI autosequencer by Sangon Biotechnology Company (Shanghai, China). All the 536 sequences were aligned using MEGA v 10.0.5 software (Kumar, Stecher, Li, Knyaz, & Tamura, 2018) and then trimmed to 782 bp, which included insertions and deletions.

#### 2.6.2 Population genetic diversity and structure analyses

To test whether patterns of genetic diversity differed between *S. canadensis* populations in the invaded (China) and native (North America) ranges, we used sequences of the chloroplast spacer regions (*psbA‐trnH* and *trnL‐F*) to estimate the number of polymorphic sites, the total number of haplotypes, haplotypic diversity (*H_d_*), and nucleotide diversity (*n*) using ARLEQUIN v 3.5.2.2 (Excoffier & Lischer, 2010). These diversity indices were computed for each population separately. We then fitted linear mixed‐effect models (in r v 3.5.2) to test whether *S. canadensis* populations from the invaded range differed significantly in mean values of the diversity indices from *S. canadensis* populations in the native range. In the models, the diversity indices were treated as dependent variables, while *S. canadensis* range (invaded vs. native) was treated as a fixed‐effect independent variable. Population identity of *S. canadensis* was treated as a random‐effect independent variable and nested within range.

To test whether *S. canadensis* populations in the invaded range had a different genetic structure than *S. canadensis* populations in the native range, we used the same sequences above to perform hierarchical analysis of molecular variance (AMOVA) (Excoffier, Smouse, & Quattro, 1992). The AMOVA was performed using the global data set to compare genetic structuring between invaded and native ranges, and hierarchically for the invaded and native ranges separately using individual populations. The hierarchical AMOVA divides the total genetic variance into components due to interindividual differences within a population and interpopulation differences within a range. Genetic differentiation between ranges and among populations within ranges was compared by the fixation index (F_ST_) and tested by AMOVA in ARLEQUIN v 3.5.2.2 (Excoffier & Lischer, 2010). Significance of genetic differentiation was tested by 1,000 random permutations. The minority cytotype exclusion hypothesis predicts that there could be a restricted gene flow between polyploid and progenitor diploid populations (Levin, 1975), which could influence genetic structure of populations that contain different cytotypes. Therefore, we also performed AMOVA based only on purely hexaploid populations that occurred in the invaded range and mixed‐ploidy populations in the native range that contained at least one hexaploid cytotype.

#### 2.6.3 Testing for population demographic expansion

Changes in demographic history can influence the frequency of alleles, the distribution of mutations, and the coalescent times of gene copies (Zhang, Edwards, Kang, & Fuller, 2012). Hence, we inferred the effects of past demographic expansion on the current genetic variation in invaded and native ranges using Tajima's D and Fu's *F_S_* neutrality tests (Fu, 1997; Tajima, 1989) and pairwise mismatch distributions between haplotypes (Rogers & Harpending, 1992). The neutrality tests were performed with DnaSP v. 6 (Rozas et al., 2017), while the mismatch distribution analyses, which test patterns of nucleotide variation against a null model expected under a sudden population expansion, were implemented in ARLEQUIN v 3.5.2.2 (Excoffier & Lischer, 2010) using 10,000 bootstrap replicates. In the neutrality tests, examination of deviation from neutrality was based on 1,000 coalescent simulations. Nonstatistical difference from zero rejects the null hypothesis of neutral evolution. Significant negative values of Tajima's D and Fu's *F_S_* indicate an excess of young or rare alleles in the genealogy, which suggest recent population expansion or purifying selection (Fu, 1997; Tajima, 1989). On the other hand, significant positive values indicate processes such as recent population bottlenecks or balancing selection (Fu, 1997; Tajima, 1989). A mismatch distribution analysis calculates various population parameters, such as the raggedness index (r), with significantly ragged populations having stable demographic histories and non‐significantly ragged populations having sudden population growth (Rogers & Harpending, 1992). For data distributed according to a sudden population expansion model (i.e., non‐significant r indices), the mismatch distribution analysis computes parameters of demographic expansion such as moment estimators of time to the expansion (Tau = 2Tu), effective population size before expansion (Theta0, θ0), and effective population size after expansion (Theta1, θ1) between the observed and expected mismatches (Rogers & Harpending, 1992). Departure from a model of sudden expansion was tested for each population by summing the squared differences (SSD) between observed and estimated mismatch distribution. To further infer the demographic history of *S. canadensis*, we determined genealogical relationships among haplotypes using the statistical parsimony algorithm implemented in ARLEQUIN v 3.5.2.2 (Excoffier & Lischer, 2010). A star‐shaped genealogy indicates a lack of (or limited) geographic structure and suggests a signature of rapid population expansion (Slatkin & Hudson, 1991). Genealogical relationships among the haplotypes were inferred using a combined data set of invasive and native haplotypes and separately for the two sets of haplotypes.

#### 2.6.4 Analysis of phylogenetic relatedness

To test whether all the 80 populations of *S. canadensis* that we sampled in the invaded and native ranges belonged to the same species (i.e., *S. canadensis*), we performed analyses of phylogenetic relatedness using sequences of 34 haplotypes that were derived from 536 individual sequences of the two chloroplast spacer regions (*psbA‐trnH* and *trnL‐F*). We applied three methods, namely the Maximum Parsimony, Maximum Likelihood, and Neighbor‐Joining. We performed Maximum Parsimony analyses with all characters equally weighted and gaps treated as missing data. For the Maximum Likelihood analyses, maximum likelihood trees were generated using the *Jukes–Cantor* model, the *Gamma distributed with the Invariant Sites (G + I)* option for the rates among sites, and *use all the sites* option for the *Gaps/Missing Data Treatment*. For the Neighbor‐Joining analysis, we applied the default settings. After 1,000 rapid bootstrap search step, bootstrap values of each node were visualized. As outgroups, we used sequences of the two chloroplast spacer regions (*psbA‐trnH* and *trnL‐F*) for *S. canadensis* seeds that we obtained from the Germplasm Resources Information Network of the United States Department of Agriculture (accession number W6 52,837) and a congener *S. decurrens*. The congener was collected from Kuocangshan Mountain in Linhai City, Zhejiang Province, China, and identified by Prof. Ming Jiang in Taizhou University, China. All phylogenetic analyses were performed with MEGA v 10.0.5 (Kumar et al., 2018). Sequences of the 34 haplotypes and the two outgroups have been submitted to the National Center for Biotechnology Information (NCBI) database (accession numbers: MN107084–MN107151, MN335287–MN335290).

## 3 RESULTS

### 3.1 Cytobiogeography of *S. canadensis*

**FIGURE 1:**
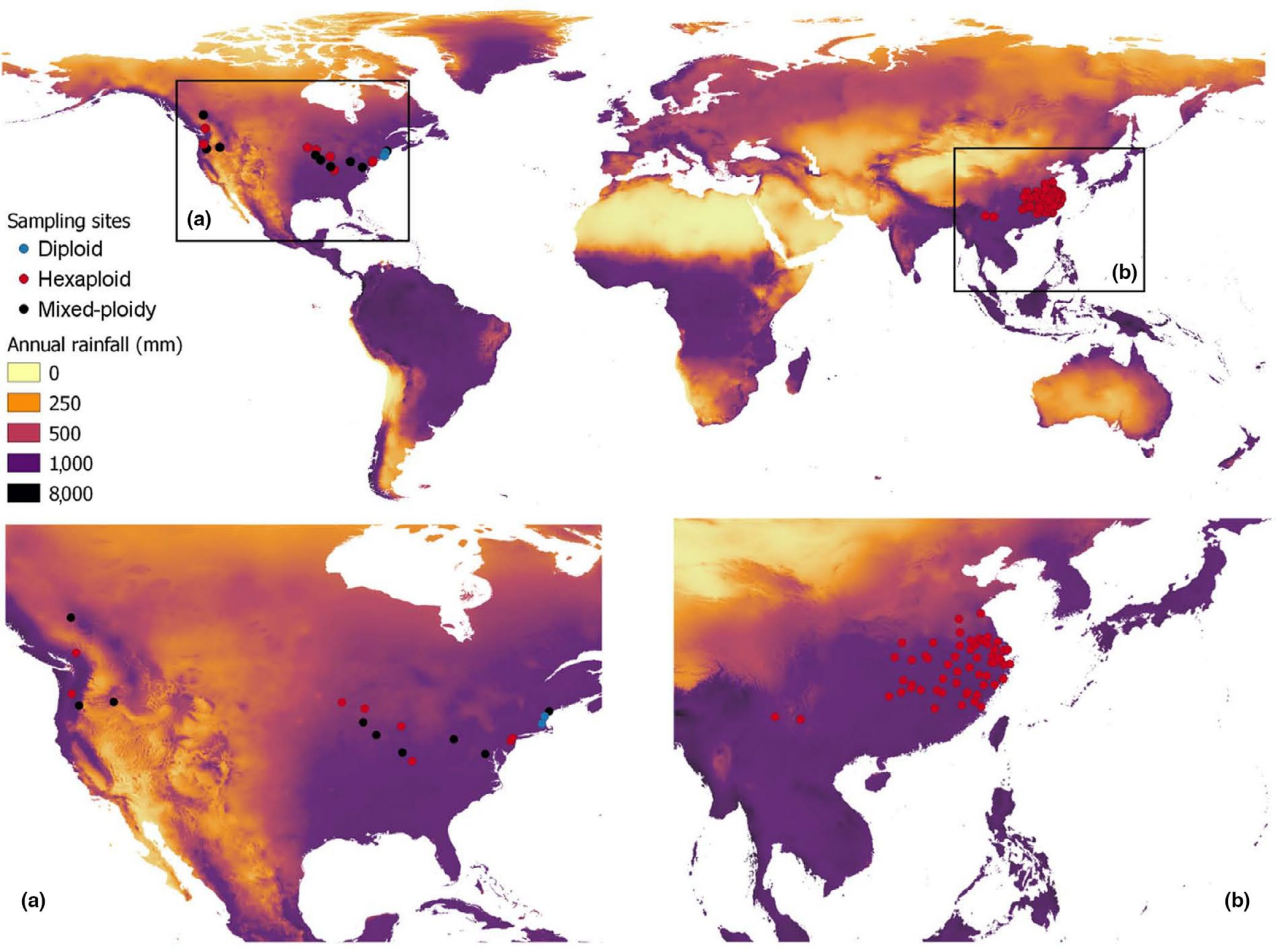
A map of the sampled populations of *Solidago canadensis* in parts of its native range in North America (a) and invaded range in China (b). Shown are the distribution of different cytotypes within the populations: Diploids are blue, hexaploids are red, while populations that exhibited mixed‐ploidy are represented by black

Among all the 80 *S. canadensis* populations that we sampled in China and North America, all invasive populations had hexaploid individuals (Figure 1 and Appendix 1). In contrast, in the native range, nine populations had a mixture of diploid and hexaploid individuals, eight populations were purely hexaploid, and three populations were purely diploid (Figure 1 and Appendix 1). Only one individual *S. canadensis* plant (from Bend City population in Oregon State, USA) was tetraploid (Appendix 1). In total, the invasive populations had 1, 474 hexaploid individuals, while the native populations had 414 hexaploid individuals (Appendix 1). There were 162 diploid individuals in the native range (Appendix 1).

### 3.2 Functional traits

**FIGURE 2:**
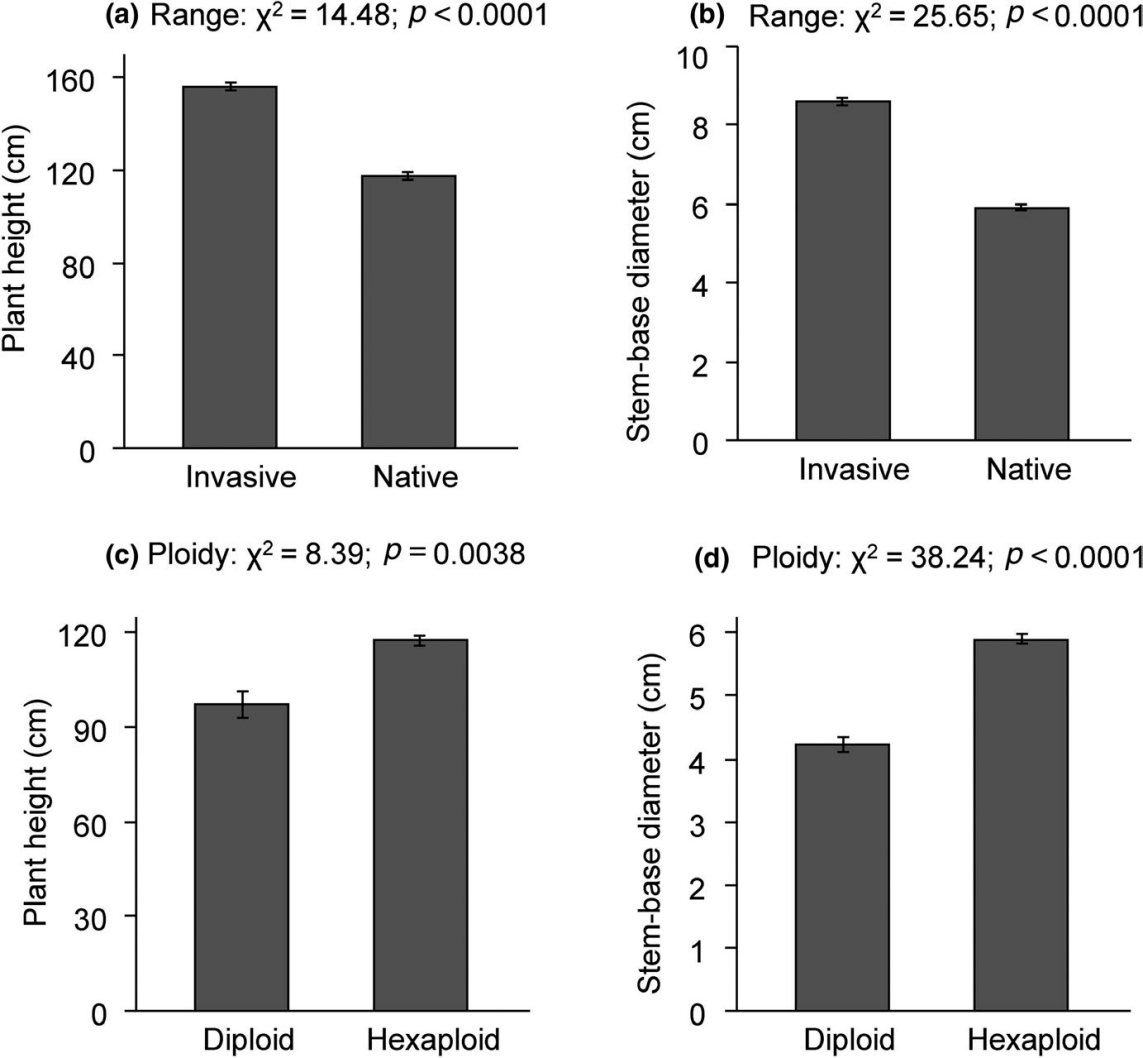
Mean (±1SE) height and stem‐base diameter of *Solidago canadensis* plants. Shown are comparisons between invasive hexaploids and native hexaploids (a & b), and between native diploids and native hexaploids (c & d)

Invasive hexaploids were significantly taller and had a greater stem‐base diameter than native hexaploids (Figure 2a & b). Similar results were found when invasive hexaploids were compared with native hexaploids from the same climatic zone (i.e., humid subtropical) (Appendix 3a & b). Native hexaploids had greater mean values of the two traits than native diploids (Figure 2c & d). Similar results were found when native hexaploids from mixed‐ploidy populations were compared with native diploids from mixed‐ploidy populations (Appendix 3c & d).

### 3.3 Climatic envelopes of *S. canadensis*

**FIGURE 3:**
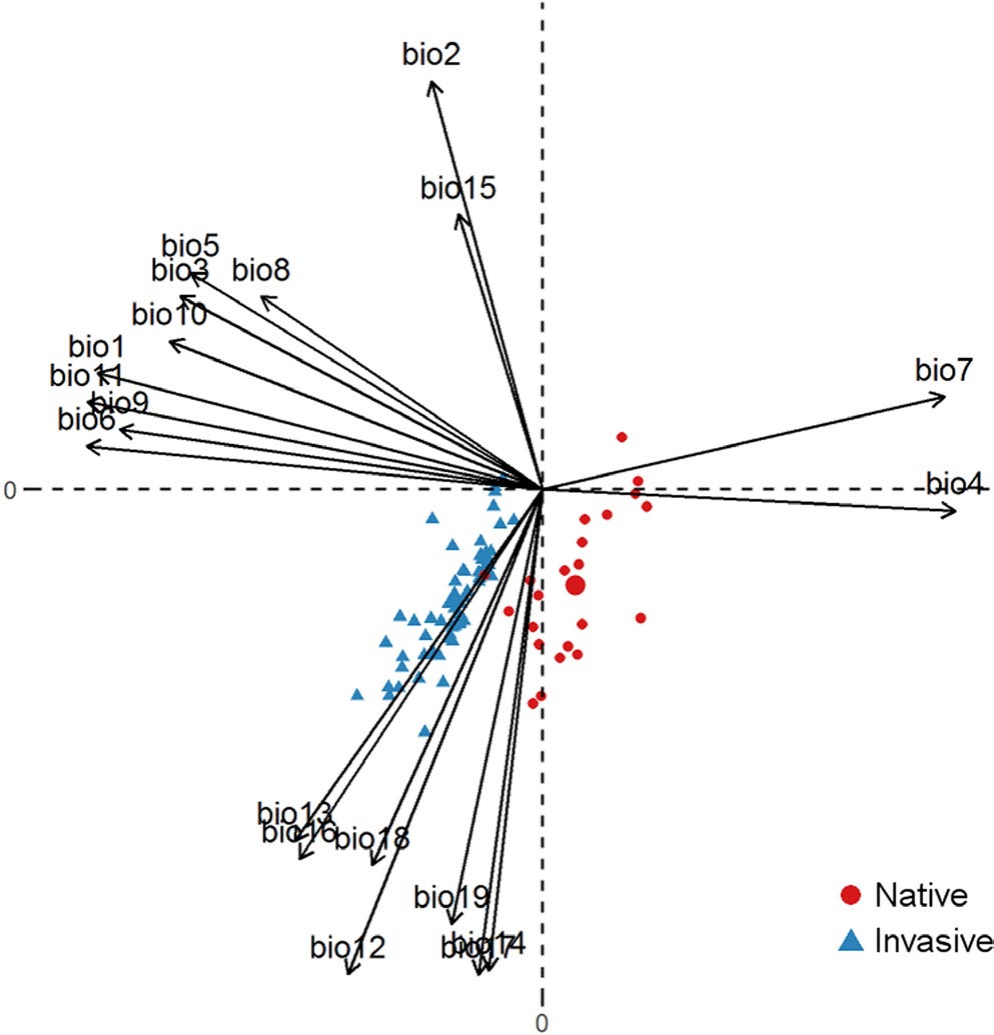
A correlation circle indicating the contribution of original climatic variables to axis1 (vertical) and axis2 (horizontal) of the principal component analysis. For variable abbreviations, see Hijmans et al. (2005). Blue triangles represent invasive populations of *S. canadensis* from China, while red circles represent *S. canadensis* populations from the native range in North America

The PCA revealed two principal components that cumulatively explained 74% of variation in the climatic data (Figure 3). The first principal component (PC1) explained 44% of the variation and reflected a precipitation gradient, while the second component (PC2) explained 30% of the variation and was correlated strongly with temperature variables (Figure 3). Independent of the ploidy level and the different sets of occurrence locations that we used in the analysis (our 80 field‐sampled populations versus 2,134 GBIF occurrence records), the climatic space occupied by invasive and native populations of *S. canadensis* was neither significantly equivalent (*p* = 1.00) nor similar (*p* > 0.14) (Table 1). In all cases, climatic envelopes clearly differentiated along the temperature gradient (PC2), with invasive populations preferring warmer and less seasonal climates (Figure 3). The invasive populations exhibited a contraction in climatic tolerance (i.e., the ratio of the climate inhabited by native populations to that by invasive populations was significantly greater than one) (Table 1). However, the invasive hexaploids and native hexaploids inhabited similar climates (*p* = 0.92 along PC1 and *p* = 0.46 along PC2) (Table 1). An analysis of a pooled data set of the 80 populations that we surveyed in China and North America did not find a significant difference in the climatic conditions among the populations (*p* = 0.74 along PC1 and *p* = 0.75 along PC2) (Table 1). In addition, diploid and hexaploid native populations occurred in similar climatic conditions (*D* = 0.78; equivalency test; *p* = 0.02; Appendix 4).

**TABLE 1:** Results of climatic envelope analysis for invasive and native populations of *Solidago canadensis*. The comparison “*Invasive *versus* native hexaploids*” compares Chinese hexaploid with North American hexaploid populations; “*Among samples*” compares variation among our field‐sampled populations in China and North America pooled together (80 populations); “*China *versus* Native range*” compares occurrences in China with North America and Mexico (our field‐sampled sites and GBIF records); “*Entire invaded range *versus* Native range*” compares occurrences in entire invaded range in China, Australia, New Zealand, and Japan with North America and Mexico (our field‐sampled sites and GBIF records). The last three comparisons did not control for a variation in ploidy level. The column “*D*” refers to the value of the Schoener's *D* metric, “*Equivalency*” to the *P* value of the equivalency test, “*Similarity*” to the *P* value of the similarity test, and “*Breadth ratio*” to the ratio between the environmental tolerance of native populations to that of invasive populations along the axes PC1 and PC2 (when a significant difference in environmental tolerance was found). For the breadth ratios, *n.s.* means that the ratio is non‐statistically different (*p > 0*.05) from one

Comparison	*D*	Equivalency	Similarity	Breadth ratio PC1	Breadth ratio PC2
Invasive versus native hexaploids	0.07	1.00	0.22	*n*.s.	*n*.s.
Among samples	0.08	1.00	0.27	*n*.s.	*n*.s.
China versus Native range	0.14	1.00	0.30	6.5	3.7
Entire invaded range versus Native range	0.20	1.00	0.14	3.6	1.5

### 3.4 Correlations between plant traits and bioclimatic variables

Stem‐base diameter had significant positive correlations with five (annual mean temperature, mean diurnal range, precipitation of the driest quarter, precipitation of the warmest quarter, and precipitation of the coldest quarter) and significant negative correlations with another five (isothermality, maximum temperature of warmest month, annual precipitation, precipitation of driest month, and precipitation of wettest quarter) bioclimatic variables (Appendix 5). On the other hand, plant height had significant positive correlations with six (annual mean temperature, mean diurnal range, temperature seasonality, mean temperature of coldest quarter, precipitation seasonality, and precipitation of coldest quarter) and significant negative correlations with four (isothermality, minimum temperature of coldest month, mean temperature of warmest quarter, and precipitation of wettest quarter) bioclimatic variables (Appendix 5).

### 3.5 Genetic diversity of *S. canadensis* in the invaded and native ranges

We found a total of 34 haplotypes from 536 sequences (289 sequences for invasive populations vs. 247 sequences for native populations) based on the two chloroplast spacer regions (*psbA‐trnH* and *trnL‐F*) (Appendix 6). The native range harbored 32 of the haplotypes, while the invaded range harbored five of the haplotypes. Haplotype H1 was the most abundant and widely distributed as it occurred in all the invasive populations and in 75% of the native populations (Appendix 6). Two other haplotypes (H9 and H11) occurred in both invaded and native ranges. The rest of the haplotypes were private, as they occurred exclusively in the invaded (H32 and H34) and native (H2‐H8, H10, H12‐H31, and H33) ranges (Appendix 6). A geographic structure in the distribution of the haplotypes is also reflected in the haplotype network, which did not have a perfect star‐like pattern in the analysis using a combined data set of invasive and native haplotypes (Appendix 7) and separate analyses for the two sets of haplotypes (results not shown). The invasive populations had a significantly lower mean number of polymorphic sites and haplotypes per population than native populations (Table 2). However, mean haplotypic and nucleotide diversities were similar between the invaded and native populations (Table 2).

**TABLE 2:** Results of linear mixed‐effect models to test whether mean population genetic diversity indices (number of polymorphic sites, mean number of haplotypes per population, haplotypic diversity (*Hd*), and nucleotide diversity (*n*)) differed significantly between invasive and native populations of *Solidago canadensis*. The indices are based on sequences of two chloroplast spacer regions (*psbA‐trnH* and *trnL‐F*; *n* = 289 individuals for invasive populations versus *n* = 247 individuals for native‐range populations). *SD* = standard deviation

Diversity index	Invaded range		Native range		*P*
	Mean	*SD*	Mean	*SD*	
Number of polymorphic sites	11.22	1.05	11.58	1.017	**0.047**
Mean number of haplotypes per population	1.54	0.56	3.62	1.203	**0.038**
Haplotypic diversity (*Hd*)	0.18	0.06	0.55	0.12	0.072
Nucleotide diversity (π)	0.028	0.015	0.029	0.015	0.250

### 3.6 Genetic structure and phylogenetic relatedness

We detected significant genetic structures at all hierarchical levels (Table 3). There was a low (7.27%) but significant genetic differentiation between invasive populations in China and native populations in North America (Table 3). There was also a significant genetic variation among all the invasive and native populations combined (40.46%), although most of the genetic variation occurred within populations (52.27%) (Table 3). Within each range, genetic divergence among populations was higher in North America (*F*_ST_ = 0.523) than in China (*F*_ST_ = 0.329) (Table 3). Nonetheless, the invasive populations exhibited higher within‐population genetic variation (67.10%) than the native populations (47.68%) (Table 3). An additional AMOVA based only on purely hexaploid populations that occurred in the invaded range and mixed‐ploidy populations in the native range that contained at least one hexaploid cytotype produced similar results (Appendix 8). A phylogenetic tree of the 34 haplotypes generated using the Maximum Likelihood, Maximum Parsimony, and Neighbor‐Joining methods revealed that clade formation received only a weak bootstrap support (Appendices 9–11). The hypothesis of recent population demographic expansions or purifying selection among the invasive populations was supported by Fu's *F_S_* test statistic (Fu's *F_S_* = −4.905; *p* = 0.007), while Tajima's D test statistic did not provide support (Tajima's D = −1.46; *p* > 0.1). However, for the native populations, both test statistics supported the hypothesis (Tajima's D = −2.43; *p* < 0.01 and Fu's *F_S_* = −8.34; *p* < 0.001). However, results of mismatch distribution analyses did not support the hypothesis of demographic expansion in the native populations (raggedness index = 0.072; *p* = 0.000 and SSD = 0.047; *p* = 0.010). A mismatch distribution is not shown for invasive populations because the least‐square procedure to fit expected‐to‐observed distributions did not converge after 2 000 steps.

**TABLE 3:** Results of a hierarchical analysis of molecular variance (AMOVA) based on two chloroplast spacer regions *(psbA‐trnH and trnL‐F)* testing for genetic variation among and within 80 populations of *Solidago canadensis* in the invaded (*n* = 289 individuals) and native (*n* = 247 individuals) ranges. The F_ST_ values are global.

Source of variation	*df*	Sum of squares	Variance components	Percentage of variation	*P*
(a) Global data set (invaded versus native range)					
Between ranges	1	435.260	1.280	7.27	0.001
Among populations	43	4,039.738	7.117	40.46	0.001
Within populations	494	4,515.257	9.196	52.27	0.001
Total	535	8,990.255	17.593		
*F*_ST_ = 0.447					
(b) Invaded range					
Among populations	23	1,437.976	4.442	32.90	0.001
Within populations	265	2,400.716	9.059	67.10	0.001
Total	288	3,838.692	13.501		
*F*_ST_ = 0.329					
(c) Native range					
Among populations	20	2,601.763	10.266	52.32	0.001
Within populations	226	2,114.541	9.356	47.68	0.001
Total	246	4,716.304	19.622		
*F*_ST_ = 0.523					

## 4 DISCUSSION

**FIGURE 4:**
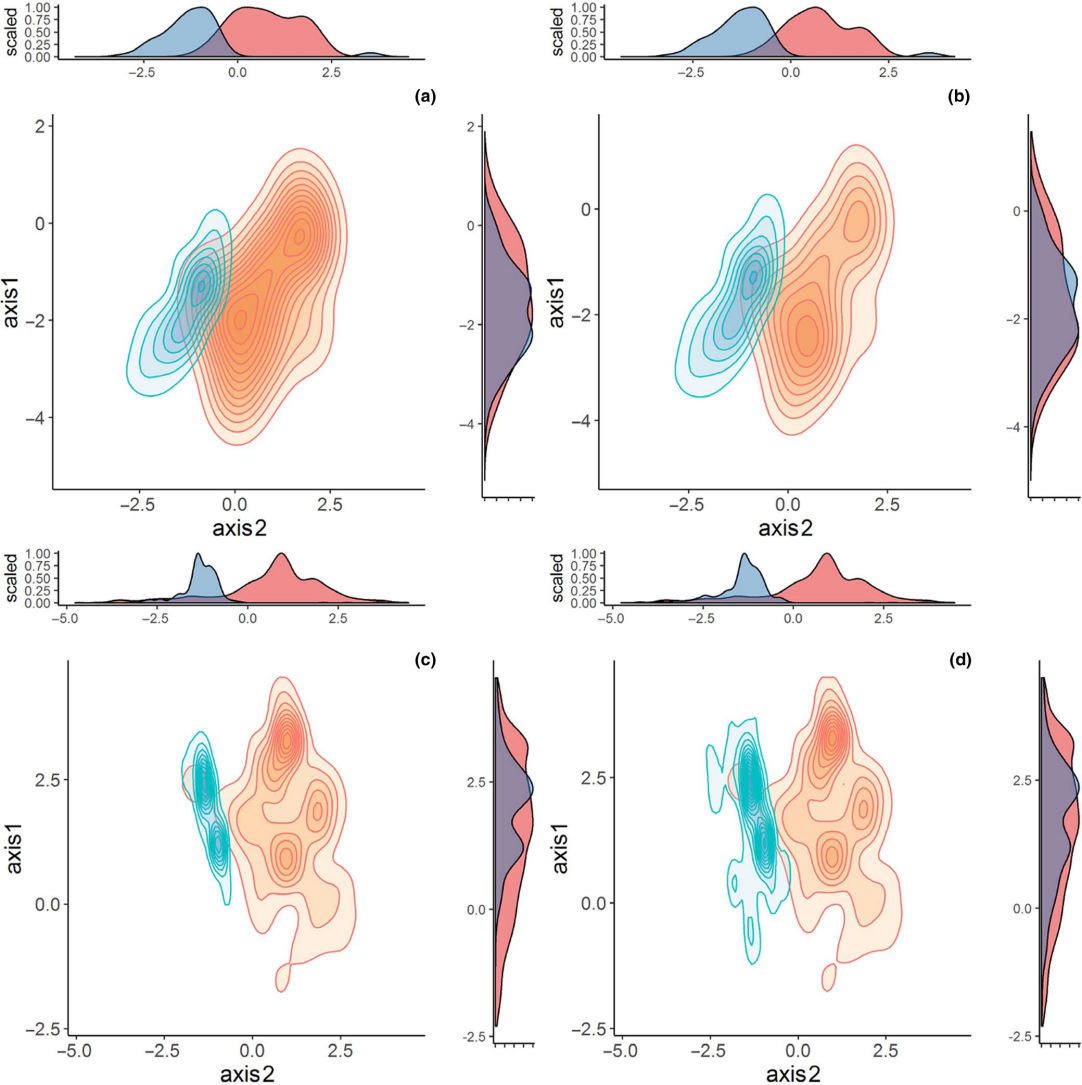
Climatic envelopes of *Solidago canadensis* in its native (orange) and invaded (blue) ranges. The following comparisons were made: (a) invasive hexaploid versus native hexaploid populations; (b) all invasive versus native populations that we sampled in the field without controlling for variation in ploidy; (c) all occurrences in North America and Mexico (our field‐sampled sites and GBIF records) versus China; (d) all occurrences in North America and Mexico versus entire invaded range in China, Australia, New Zealand, and Japan. Histograms on top and on the right side of each figure give the standardized density distribution along the first (PC1) and second (PC2) principal components, respectively

The present study used *S. canadensis* to test whether invasive populations had a higher proportion of polyploids, were significantly larger, and occupied a wider range of climatic conditions than conspecific native‐range populations. Because biogeographic differences between invasive and native populations in cytotype frequency and functional traits could be due to stochastic founder events, the study also tested whether the invasive populations had overcome (or minimized) founder effects. We found that all invasive *S. canadensis* populations sampled in China were purely hexaploid, while the native populations sampled in North America were diploid, tetraploid, and hexaploid. The invasive hexaploids were significantly taller and had a  larger stem‐base diameter than native hexaploids. Within the native range, hexaploid *S. canadensis* plants were significantly taller and had a larger stem‐base diameter than conspecific diploids. Climatic envelope assessment found that invasive and native populations occupied different climatic conditions, with invasive populations occurring in warmer and less seasonal climates than native populations. However, there was no significant correlation between ploidy level and the climatic envelope of *S. canadensis*, at least along the two axes of the PCA that cumulatively explained 74% of variation in climate. Our results are robust to a sampling bias because two comparisons involving a global data set of *S. canadensis* occurrence records versus a part of invaded range in China and the global data set versus the entire invaded range produced similar results (Table 1 and Figure 4). Molecular phylogeography data provide signatures of reduced genetic founder effects in the invaded range, likely as a result of multiple introduction events. Taken together, these results suggest that ploidy level does not influence climatic space of *S. canadensis* and that other factors may determine its invasiveness in China.

### 4.1 Cytobiogeography and functional trait differences

The present findings that invasive *S. canadensis* populations in China were comprised only of hexaploid individuals, while native populations in North America were diploid, tetraploid, and hexaploid, could be explained by three nonmutually exclusive hypotheses. First, it is plausible that only hexaploid plants were able to adapt to new conditions after introduction and compete with native species, while other cytotypes failed. Second, because *S. canadensis* was introduced to China as an ornamental garden plant (Xu et al., 2012; Zhao et al., 2015), it is possible gardeners preferentially introduced hexaploids that had some unique desirable traits like larger sizes. Third, because invasive plant introductions are often stochastic events, strong founder events may have caused a loss of cytotype diversity in the invaded range (Mráz et al., 2014). This may occur particularly when there is a spatial separation of the different cytotypes in the native range, which increases the chance that only a subset of the cytotypic diversity is introduced (Kubatova et al., 2008; Lafuma, Balkwill, Imbert, Verlaque, & Maurice, 2003). However, the second and third scenarios seem less plausible because our molecular phylogeography data (Tables 2, 3 and Appendix 8) and previous studies (Xu et al., 2012; Zhao et al., 2015) suggest that the Chinese populations have minimized founder effects through multiple introductions.

The present finding that invasive *S. canadensis* hexaploids had greater height and stem‐base diameter than native hexaploids (Figure 2) and that the two traits were significantly correlated with various bioclimatic factors negatively and positively (Appendix 5) suggests that divergence in the trait means could be the outcome of differential natural selection imposed by the climatic factors. Evolution of increased sizes has been associated with invasiveness of several plant species (e.g., Oduor et al., 2013; Whitney & Gabler, 2008; Oduor, Stift, & van Kleunen, 2015a; Oduor, van Kleunen, & Stift, 2017 ). Other studies that compared traits of invasive polyploids and conspecific native‐range polyploids in common‐garden settings produced mixed results. For instance, the invasive *Oxalis pes‐caprae* tetraploids produced more aboveground biomass and offspring bulbs than native *O. pes‐caprae* tetraploids (Tavares et al., 2019). Within *S. gigantea*, invasive tetraploids produced more biomass than native‐range hexaploids (Nagy et al., 2018). In contrast, invasive tetraploid *C. stoebe* had similar length and number of florets, length of capitula and longest branch, leaf weight and leaf area as conspecific native tetraploids (Mráz, 2011). In *Vicia cracca*, invasive tetraploids had greater seedling height and aboveground biomass than native tetraploids; however, the two groups of plants produced similar numbers of seeds and branches (Líblová, Eliášová, & Münzbergová, 2017). Biogeographical comparisons of traits that use small and nonrepresentative subsets of populations may not give a complete picture of the ecological and evolutionary processes that cause trait divergence between invaded and native ranges (Rosche et al., 2019). Moreover, comparisons of plant traits between ranges through in situ field measurements can give different results from common‐garden experiments (Rosche et al., 2019). Therefore, more comprehensive studies that span the entire invaded and native ranges of *S. canadensis* and combine manipulative common‐garden experiments with cytobiogeography and molecular phylogeography are required to more rigorously test whether divergence in ploidy levels and functional traits between ranges could be due to natural selection.

### 4.2 No correlation between ploidy level and climatic envelope of *S. canadensis*

The invasive *S. canadensis* populations occupied a novel climatic envelope (i.e., there was no significant envelope overlap with native *S. canadensis*) and exhibited a contraction in climatic tolerance (i.e., occurred in warmer and less seasonal climates) relative to native *S. canadensis* populations independent of variation in ploidy level (Table 1; Figures 3 and 4). Within the native range, diploid and hexaploid *S. canadensis* populations occurred in similar climatic conditions. While our sampling of *S. canadensis* cytotype distribution was limited to only parts of the invaded (China) and native (North America) ranges, with the invaded range dominated by one climatic zone, the present results suggest that polyploidy does not influence *S. canadensis* climatic tolerance. Future studies that cover the entire climatic ranges of the species are required to validate our findings.

### 4.3 Population demographic expansions in both ranges

Results of Tajima's D and Fu's *F_S_* neutrality tests suggest that either purifying selection acted on the *S. canadensis* populations or the populations experienced recent demographic expansions. However, because the two chloroplast spacer regions (*psbA‐trnH* and *trnL‐F*) are non‐coding loci and are thus unlikely to be under selection, these results more likely indicate population demographic expansions. Although the Fu's *F_S_* neutrality test was significant for invasive populations, while Tajima's *D* was not, it has been shown that Fu's *F_S_* test is more powerful than Tajima's *D* (Ramos‐Onsins & Rozas, 2002). This suggests that population demographic expansion occurred in the invaded range as well.

### 4.4 Signatures of reduced genetic founder effects in the invaded range

The finding that invasive populations had significantly lower mean number of polymorphic sites and haplotypes than native populations (Table 2), and the AMOVA results showing genetic differentiation between invaded and native ranges (Tables 3; Appendix 8) suggest some degree of founder effect in the invaded range. Nevertheless, it is likely that the founder effect is not strong, likely as a result of multiple introduction events. Multiple introductions from distinct genetic sources may help to overcome founder effects (Novak & Mack, 1993). There are four lines of evidence supporting the likely scenario of multiple introduction events. First, there were similar levels of nucleotide and haplotypic diversities between the invaded and native ranges (Table 2). Second, the AMOVA results showing that a larger percentage of the total variance in the invaded range is harbored within populations compared to the lower percent of total variance harbored within populations in the native range suggest that populations are more clearly differentiated in the native range than in the invaded range (Table 3). It is likely that multiple introduction episodes from different populations in the native range converted the high level of among‐population genetic variation in the native range to high within‐population genetic variation in the introduced range. Third, there was a novel co‐occurrence of two haplotypes H9 and H11 in invasive populations in Taizhou that otherwise occurred in allopatric native‐range populations in Massachusetts, Iowa, Illinois, Michigan, New Jersey, Wisconsin, and Rhode Island (Appendix 6 and Appendix 7). Thus, it is possible that pervasive movement of genetic materials in the invaded range following introduction from multiple sources contributed to a reduction in population differentiation and genetic bottleneck. Fourth, demographic expansions that we found to have occurred in the invaded range could also have contributed to a reduction in genetic founder effects. Demographic and range expansion are expected to generate an excess of recent, and thus rare, mutations. As *S. canadensis* has been present in China for at least 80 years, it is plausible that over multiple generations, mutations increased genetic diversity in the invaded range.

### 4.5 All sampled populations belonged to *S. canadensis*

Although the ecological envelope of *S. canadensis* can overlap with that of its congeners like *S. gigantea* and *S. altissima* in the native range, our analysis of phylogenetic relatedness among the study populations did not reveal any clear pattern of clade formation (i.e., all populations were monophyletic; Appendices 9–11). Thus, it is highly likely that the invasive and native populations that we sampled in the field all belonged to *S. canadensis*.

## 5 CONCLUSION

Our results suggest that polyploidy does not influence climatic tolerance of *S. canadensis* in its invaded and native ranges. While a caveat exists for our study because our sampling of *S. canadensis* cytobiogeography did not cover its entire invaded and native ranges, the results instead indicate that unknown genetic and environmental factors may influence *S. canadensis* climatic tolerance. Our results provide a useful first step to comprehensively test whether polyploidy influences climatic envelope of *S. canadensis*. For *S. canadensis*, effects of polyploidy at molecular and nuclear levels have not been investigated and would be a first step in determining how genetic changes scale up to physiological processes and ultimately biogeographic distribution patterns. Therefore, future mechanistic studies should couple cytobiogeographical and molecular phylogeography approaches with functional genomic approaches that document gene expression patterns under different ecological conditions. In addition, experimental studies such as multisite common‐garden and reciprocal transplants in both invaded and native ranges will be required.

## CONFLICT OF INTEREST

The authors declare that there is no conflict of interest.

## AUTHOR CONTRIBUTION

**Jizhong Wan:** Conceptualization (equal); Data curation (equal); Formal analysis (equal); Methodology (equal); Software (equal); Writing‐original draft (equal); Writing‐review & editing (supporting). **Ayub M. O. Oduor:** Conceptualization (equal); Data curation (equal); Formal analysis (equal); Methodology (equal); Software (equal); Validation (equal); Writing‐review & editing (lead). **Robin Pouteau:** Conceptualization (supporting); Data curation (equal); Formal analysis (equal); Methodology (equal); Software (equal); Validation (equal); Writing‐review & editing (equal). **Beilei Wang:** Data curation (supporting); Formal analysis (supporting); Investigation (supporting); Methodology (supporting). **Luxi Chen:** Data curation (equal); Investigation (equal). **Beifen Yang:** Data curation (equal); Investigation (equal). **Feihai Yu:** Conceptualization (supporting); Writing‐review & editing (supporting). **Junmin Li:** Conceptualization (lead); Funding acquisition (lead); Investigation (lead); Methodology (lead); Project administration (lead); Validation (lead); Writing‐original draft (lead); Writing‐review & editing (lead).

## Supporting information

Appendix S1‐S11

## Data Availability

DNA sequences have been deposited in the National Center for Biotechnology Information (NCBI) database (accession numbers: MN107084–MN107151, MN335287–MN335290). The data that support the findings of this study have been deposited in Dryad with doi:https://doi.org/10.5061/dryad.37pvmcvgb.
