# Supplementary material for: Can polyploidy confer invasive plants with a wider climatic tolerance? A test using Solidago canadensis
Source: Ecol Evol. 2020 May 26;10(12):5617–30. doi: 10.1002/ece3.6303 (PMC7319170; doi:10.1002/ece3.6303)
Supplement: Supplementary file 1 — Appendix S1‐S11 [file ECE3-10-5617-s001.doc]

**Appendix 1.** Location, sample size and cytotype of *Solidago canadensis* populations that were sampled in the invaded range in China and native range in North America.

| Population | Range | Cytotype | Sample size (n) | Longitude | Latitude |
| --- | --- | --- | --- | --- | --- |
| Fuzhou City, Fujian Province, China | Invaded | Hexaploid | 12 | 119.359E | 26.098N |
| Wuyi City, Fujian Province, China | Invaded | Hexaploid | 30 | 118.010E | 27.230N |
| Ningde City, Fujian Province, China | Invaded | Hexaploid | 30 | 118.74E | 26.560N |
| Nanping City, Fujian Province, China | Invaded | Hexaploid | 30 | 118.980E | 27.180N |
| Sanming City, Fujian Province, China | Invaded | Hexaploid | 21 | 117.560E | 26.240N |
| Jianyang City, Fujian Province, China | Invaded | Hexaploid | 16 | 118.090E | 27.410N |
| Minhang District, Shanghai City, China | Invaded | Hexaploid | 12 | 121.433E | 31.307N |
| Pudong District, Shanghai City, China | Invaded | Hexaploid | 12 | 121.804E | 31.354N |
| Jingdezhen City, Jiangxi Province, China | Invaded | Hexaploid | 12 | 117.166E | 29.318N |
| Jiujiang City, Jiangxi Province, China | Invaded | Hexaploid | 12 | 116.283E | 29.985N |
| Yingtan City, Jiangxi Province, China | Invaded | Hexaploid | 30 | 117.053E | 28.272N |
| Nanchang City, Jiangxi Province, China | Invaded | Hexaploid | 30 | 115.814E | 28.664N |
| Ganzhou City, Jiangxi Province, China | Invaded | Hexaploid | 30 | 115.059E | 25.929N |
| Pingxiang City, Jiangxi Province, China | Invaded | Hexaploid | 30 | 113.764E | 27.647N |
| Ji’an City, Jiangxi Province, China | Invaded | Hexaploid | 30 | 115.298E | 27.595N |
| Fuzhou City, Jiangxi Province, China | Invaded | Hexaploid | 30 | 115.734E | 27.384N |
| Xinyang City, Henan Province, China | Invaded | Hexaploid | 30 | 114.916E | 32.001N |
| Yiyang City, Hunan Province, China | Invaded | Hexaploid | 30 | 112.140E | 28.520N |
| Changsha City, Hunan Province, China | Invaded | Hexaploid | 30 | 112.950E | 28.140N |
| Shaoyang City, Hunan Province, China | Invaded | Hexaploid | 30 | 110.840E | 27.040N |
| Loudi City, Hunan Province, China | Invaded | Hexaploid | 14 | 111.980E | 27.420N |
| Xiangtan City, Hunan Province, China | Invaded | Hexaploid | 30 | 112.880E | 27.850N |
| HangzhouCity, Zhejiang Province, China | Invaded | Hexaploid | 12 | 120.297E | 30.161N |
| Taizhou City, Zhejiang Province | Invaded | Hexaploid | 12 | 121.397E | 28.656N |
| Wenzhou City, Zhejiang Province | Invaded | Hexaploid | 12 | 120.607E | 28.126N |
| Ningbo City 1, Zhejiang Province, China | Invaded | Hexaploid | 30 | 121.456E | 29.950N |
| Ningbo City 2, Zhejiang Province, China | Invaded | Hexaploid | 30 | 121.180E | 30.360N |
| Ningbo City 3, Zhejiang Province, China | Invaded | Hexaploid | 30 | 121.176E | 30.360N |
| Jinhua City, Zhejiang Province, China | Invaded | Hexaploid | 30 | 119.870E | 28.930N |
| Huzhou City, Zhejiang Province, China | Invaded | Hexaploid | 30 | 120.399E | 30.740N |
| Quzhou City, Zhejiang Province, China | Invaded | Hexaploid | 30 | 119.171E | 29.060N |
| Zhoushan City, Zhejiang Province, China | Invaded | Hexaploid | 30 | 121.998E | 30.050N |
| Jiaxing City, Zhejiang Province, China | Invaded | Hexaploid | 30 | 121.027E | 30.600N |
| Shaoxing City, Zhejiang Province, China | Invaded | Hexaploid | 30 | 120.516E | 30.150N |
| Lishui City, Zhejiang Province, China | Invaded | Hexaploid | 30 | 119.594E | 28.098N |
| Lianyungang City, Jiangsu Province, China | Invaded | Hexaploid | 12 | 119.331E | 34.711N |
| Nanjing City, Jiangsu Province, China | Invaded | Hexaploid | 12 | 118.849E | 31.784N |
| Nantong City, Jiangsu Province, China | Invaded | Hexaploid | 12 | 120.842E | 32.072N |
| Xuzhou City, Jiangsu Province, China | Invaded | Hexaploid | 30 | 117.295E | 34.197N |
| Changzhou City, Jiangsu Province, China | Invaded | Hexaploid | 30 | 119.938E | 31.878N |
| Yangzhou City, Jiangsu Province, China | Invaded | Hexaploid | 30 | 119.159E | 32.377N |
| Taizhou City, Jiangsu Province, China | Invaded | Hexaploid | 30 | 119.974E | 32.529N |
| Suzhou City, Jiangsu Province, China | Invaded | Hexaploid | 30 | 120.583E | 31.336N |
| Zhenjiang City 1, Jiangsu Province, China | Invaded | Hexaploid | 30 | 119.404E | 32.232N |
| Zhenjiang City 2, Jiangsu Province, China | Invaded | Hexaploid | 30 | 119.497E | 32.236N |
| Zhenjiang City 3, Jiangsu Province, China | Invaded | Hexaploid | 30 | 119.573E | 32.164N |
| Wuhan City 1, Hubei Province, China | Invaded | Hexaploid | 12 | 114.218E | 30.782N |
| Wuhan City 2, Hubei Province, China | Invaded | Hexaploid | 12 | 114.415E | 30.543N |
| Qianjiang City, Hubei Province, China | Invaded | Hexaploid | 13 | 112.936E | 30.394N |
| Xiangyang City, Hubei Province, China | Invaded | Hexaploid | 5 | 112.039E | 31.994N |
| Yichang City, Hubei Province, China | Invaded | Hexaploid | 7 | 111.361E | 30.662N |
| Wuhu City 1, Anhui Province, China | Invaded | Hexaploid | 12 | 118.387E | 31.342N |
| Wuhu City 2, Anhui Province, China | Invaded | Hexaploid | 30 | 118.368E | 31.384N |
| Chuzhou City, Anhui Province, China | Invaded | Hexaploid | 30 | 118.402E | 32.197N |
| Hefei City, Anhui Province, China | Invaded | Hexaploid | 30 | 117.379E | 31.728N |
| Anqing City, Anhui Province, China | Invaded | Hexaploid | 30 | 116.949E | 30.712N |
| Xuancheng City, Anhui Province, China | Invaded | Hexaploid | 30 | 118.974E | 30.613N |
| Huangshan City, Anhui Province, China | Invaded | Hexaploid | 30 | 118.285E | 29.728N |
| Bengbu City, Anhui Province, China | Invaded | Hexaploid | 30 | 117.405E | 32.936N |
| Kunming City, Yunnan Province, China | Invaded | Hexaploid | 30 | 102.650E | 24.910N |
| Guilin City, Guangxi Province, China | Invaded | Hexaploid | 30 | 100.288E | 25.157N |
| Bend City, Oregon State, USA | Native | Hexaploid | 3 | 121.771W | 44.280N |
|  |  | Tetraploid | 1 |  |  |
|  |  | Diploid | 29 |  |  |
| John day City, Oregon State, USA | Native | Hexaploid | 7 | 117.978W | 44.671N |
|  |  | Diploid | 24 |  |  |
| Portland City, Oregon State, USA | Native | Hexaploid | 30 | 122.574W | 45.563N |
| Saint Paul City, Minnesota, USA | Native | Hexaploid | 30 | 92.998W | 44.627N |
| Wilton City, Wisconsin, USA | Native | Hexaploid | 32 | 90.505W | 43.953N |
| Dubuque City, Iowa, USA | Native | Hexaploid | 28 | 90.681W | 42.448N |
|  |  | Diploid | 5 |  |  |
| Roberts City, Illinois, USA | Native | Hexaploid | 26 | 89.246W | 41.062N |
|  |  | Diploid | 4 |  |  |
| Shelby City, Kentucky State, USA | Native | Hexaploid | 30 | 85.330W | 38.204N |
| Monroe City, Indiana State, USA | Native | Hexaploid | 29 | 86.386W | 39.151N |
|  |  | Diploid | 1 |  |  |
| Salem City, Ohio State, USA | Native | Hexaploid | 29 | 80.757W | 40.598N |
|  |  | Diploid | 2 |  |  |
| Benton Habor City, Michigan State, USA | Native | Hexaploid | 30 | 86.518W | 42.031N |
| Portland City, Maine State, USA | Native | Diploid | 31 | 70.319W | 43.653N |
|  |  | Hexaploid | 18 |  |  |
| Portsmouth City, Rhode Island State, USA | Native | Diploid | 32 | 70.808W | 43.098N |
| Boston City, Massachusetts State, USA | Native | Diploid | 31 | 71.121W | 42.298N |
| Trenton City, New Jersey State, USA | Native | Hexaploid | 29 | 74.530W | 40.420N |
| Newark City, New Jersey State, USA | Native | Hexaploid | 7 | 74.335W | 40.733N |
| Dranesville City, Virginia State, USA | Native | Hexaploid | 30 | 77.316W | 38.983N |
|  |  | Diploid | 1 |  |  |
| Prince George, British Columbia, Canada | Native | Hexaploid | 23 | 122.67W | 53.883N |
|  |  | Diploid | 2 |  |  |
| Aleza Lake Research Forest, British Columbia, Canada | Native | Hexaploid | 33 | 122.1W | 50.04N |

**Appendix 2**. The sequences of four universal primers for two chloroplast spacer regions (*psbA-trnH* and *trnL-F*) that were used to investigate phylogeography of, and phylogenetic relatedness among, *Solidago canadensis* populations in the invaded range in China and native range in North America.

| Spacer region | Primer | Sequence（5’ 3’） |
| --- | --- | --- |
| *psbA-trnH* | psbA3'f  trnHf | GTTATGCATGAACGTAATGCTC  CGCGCATGGTGGATTCACAATCC |
| *trnL-F* | e  f | GGTTCAAGTCCCTCTATCCC  ATTTGAACTGGTGACACGAG |

**
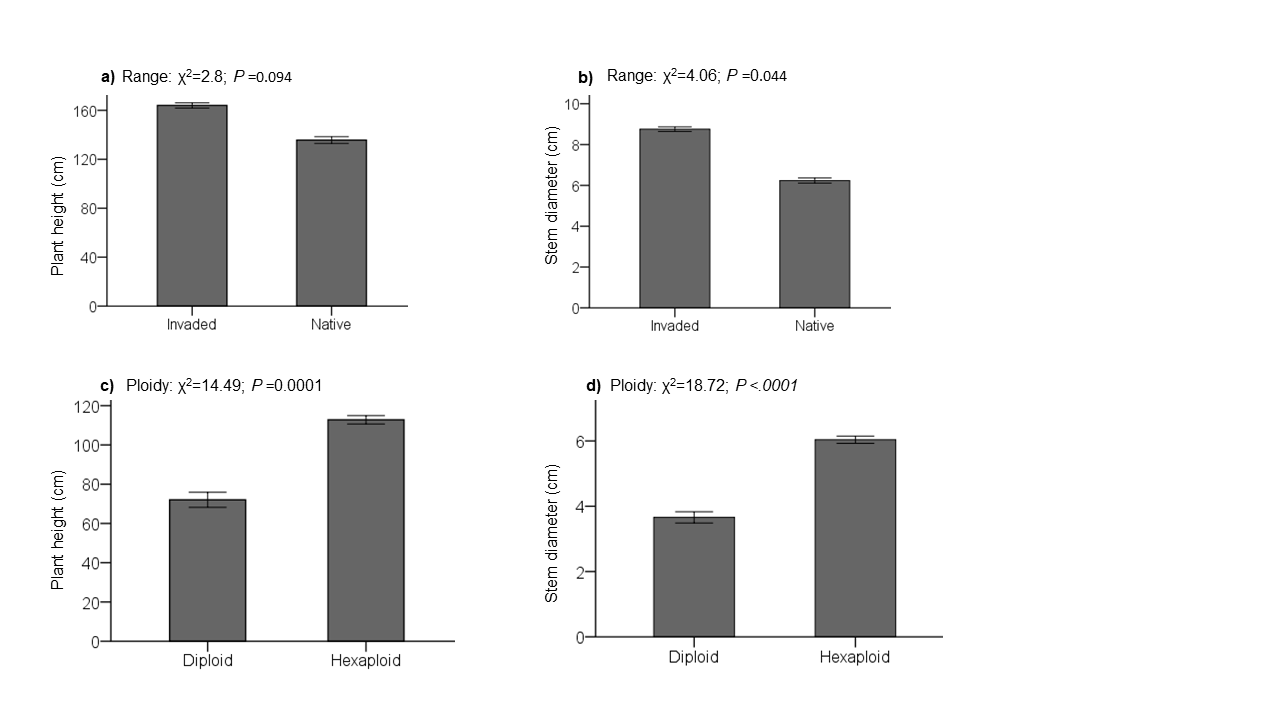
**

**Appendix 3**Mean (±1SE) height and stem-base diameter of *Solidago canadensis* plants. Shown are the comparisons between invasive hexaploid (n=1025) and native hexaploid (n=99) plants that were all sampled from humid subtropical climate (**a** & **b**), and between native diploid plants (n=91) and native hexaploid plants (n=196) that were sampled in populations with mixed-ploidy levels (**c** & **d**).


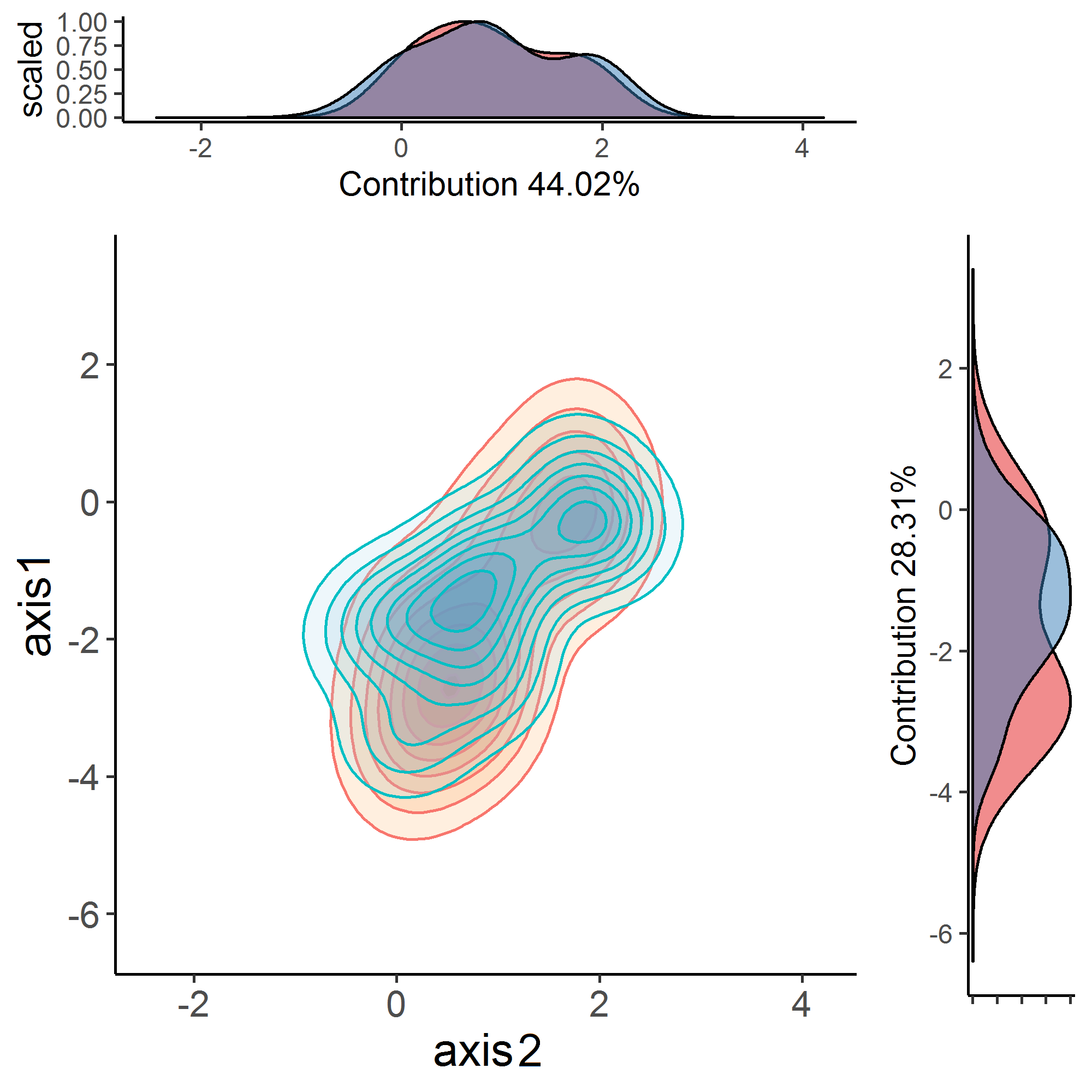


**Appendix 4.** Climatic envelope of native populations of *Solidago canadensis* according to their ploidy level (orange = diploids; blue = hexaploids). Histograms on top and on the right side of the figure give the standardized density distribution along the first (PC1) and second (PC2) principal components, respectively.

**Appendix 5.** Results of multiple linear regressions testing for a correlation between stem - base diameter and height of *Solidago canadensis* plants and bioclimatic variables.

|  | **Stem - base diameter (R^2^ adjusted=0.37)** | | | | | **Plant height (R^2^ adjusted=0.236)** | | | |
| --- | --- | --- | --- | --- | --- | --- | --- | --- | --- |
| Description of BIOs |  | Estimate | Std  error | t | *P* | Estimate | Std error | t | *P* |
|  | (Intercept) | 19.75 | 4.35 | 4.54 | **<0.0001** | 159.45 | 85.01 | 1.876 | 0.061 |
| Annual Mean Temperature | bio1 | 0.17 | 0.08 | 2.07 | **0.038** | 6.60 | 1.44 | 4.60 | **<0.0001** |
| Mean Diurnal Range | bio2 | 0.19 | 0.05 | 4.07 | **<0.0001** | 1.77 | 0.83 | 2.13 | **0.034** |
| Isothermality | bio3 | -0.75 | 0.13 | -5.61 | **<0.0001** | -10.05 | 2.49 | -4.04 | **<0.0001** |
| Temperature Seasonality | bio4 | 0.00 | 0.00 | 1.26 | 0.207 | 0.11 | 0.04 | 2.75 | **0.0060** |
| Max Temperature of Warmest Month | bio5 | -0.12 | 0.03 | -3.90 | **<0.0001** | -0.61 | 0.56 | -1.08 | 0.28 |
| Min Temperature of Coldest Month | bio6 | -0.04 | 0.03 | -1.19 | 0.235 | -2.34 | 0.58 | -4.07 | **<0.0001** |
| Mean Temperature of Wettest Quarter | bio8 | 0.00 | 0.00 | -0.13 | 0.899 | -0.10 | 0.08 | -1.37 | 0.171 |
| Mean Temperature of Driest Quarter | bio9 | 0.00 | 0.00 | 0.61 | 0.543 | -0.07 | 0.06 | -1.31 | 0.192 |
| Mean Temperature of Warmest Quarter | bio10 | -0.10 | 0.09 | -1.06 | 0.289 | -7.37 | 1.73 | -4.27 | **<0.0001** |
| Mean Temperature of Coldest Quarter | bio11 | 0.14 | 0.10 | 1.39 | 0.165 | 5.09 | 1.74 | 2.92 | **0.0035** |
| Annual Precipitation | bio12 | -0.01 | 0.00 | -4.43 | **<0.0001** | 0.02 | 0.04 | 0.37 | 0.710 |
| Precipitation of Wettest Month | bio13 | 0.02 | 0.01 | 1.85 | 0.065 | 0.12 | 0.16 | 0.78 | 0.437 |
| Precipitation of Driest Month | bio14 | -0.12 | 0.03 | -4.21 | **<0.0001** | 0.53 | 0.55 | 0.95 | 0.340 |
| Precipitation Seasonality | bio15 | 0.07 | 0.03 | 2.53 | 0.0115 | 1.93 | 0.43 | 4.52 | **<0.0001** |
| Precipitation of Wettest Quarter | bio16 | -0.01 | 0.00 | -3.22 | **0.0013** | -0.47 | 0.07 | -6.53 | **<0.0001** |
| Precipitation of Driest Quarter | bio17 | 0.04 | 0.01 | 3.96 | **<0.0001** | -0.09 | 0.20 | -0.46 | 0.646 |
| Precipitation of Warmest Quarter | bio18 | 0.02 | 0.00 | 4.13 | **<0.0001** | 0.10 | 0.07 | 1.44 | 0.149 |
| Precipitation of Coldest Quarter | bio19 | 0.03 | 0.01 | 6.11 | **<0.0001** | 0.44 | 0.09 | 4.74 | **<0.0001** |

**Appendix 6**. Distribution of 34 haplotypes of *Solidago canadensis* in the invaded and native ranges

| Population code* | Range | H1 | H2 | H3 | H4 | H5 | H6 | H7 | H8 | H9 | H10 | H11 | H12 | H13 | H14 | H15 | H16 | H17 | H18 | H19 | H20 | H21 | H22 | H23 | H24 | H25 | H26 | H27 | H28 | H29 | H30 | H31 | H32 | H33 | H34 |
| --- | --- | --- | --- | --- | --- | --- | --- | --- | --- | --- | --- | --- | --- | --- | --- | --- | --- | --- | --- | --- | --- | --- | --- | --- | --- | --- | --- | --- | --- | --- | --- | --- | --- | --- | --- |
| BB | Invaded | 13 |  |  |  |  |  |  |  |  |  |  |  |  |  |  |  |  |  |  |  |  |  |  |  |  |  |  |  |  |  |  |  |  |  |
| BC | Native | 3 | 2 | 2 | 2 | 1 | 1 |  |  |  |  |  |  |  |  |  |  |  |  |  |  |  |  |  |  |  |  |  |  |  |  |  |  |  |  |
| CS | Invaded | 9 |  |  |  |  |  |  |  | 3 |  |  |  |  |  |  |  |  |  |  |  |  |  |  |  |  |  |  |  |  |  |  |  |  |  |
| FZ | Invaded | 10 |  |  |  |  |  |  |  | 2 |  |  |  |  |  |  |  |  |  |  |  |  |  |  |  |  |  |  |  |  |  |  |  |  |  |
| GL | Invaded | 7 |  |  |  |  |  |  |  | 1 |  |  |  |  |  |  |  |  |  |  |  |  |  |  |  |  |  |  |  |  |  |  |  |  |  |
| GS | Invaded | 14 |  |  |  |  |  |  |  |  |  |  |  |  |  |  |  |  |  |  |  |  |  |  |  |  |  |  |  |  |  |  |  |  |  |
| HK | Invaded | 10 |  |  |  |  |  |  |  |  |  |  |  |  |  |  |  |  |  |  |  |  |  |  |  |  |  |  |  |  |  |  |  |  |  |
| HQ | Invaded | 12 |  |  |  |  |  |  |  |  |  |  |  |  |  |  |  |  |  |  |  |  |  |  |  |  |  |  |  |  |  |  |  |  |  |
| HZ | Invaded | 12 |  |  |  |  |  |  |  |  |  |  |  |  |  |  |  |  |  |  |  |  |  |  |  |  |  |  |  |  |  |  |  |  |  |
| IA | Native | 5 |  |  |  |  |  |  | 4 |  | 1 | 1 | 1 |  |  |  |  |  |  |  |  |  |  |  |  |  |  |  |  |  |  |  |  |  |  |
| IL | Native | 7 |  |  |  |  |  |  | 1 |  | 1 | 2 |  | 1 |  |  |  |  |  |  |  |  |  |  |  |  |  |  |  |  |  |  |  |  |  |
| IN | Native | 5 |  |  |  |  |  |  | 1 |  |  | 5 |  |  | 1 |  |  |  |  |  |  |  |  |  |  |  |  |  |  |  |  |  |  |  |  |
| JDZ | Invaded | 5 |  |  |  |  |  |  |  | 7 |  |  |  |  |  |  |  |  |  |  |  |  |  |  |  |  |  |  |  |  |  |  |  |  |  |
| JJ | Invaded | 12 |  |  |  |  |  |  |  |  |  |  |  |  |  |  |  |  |  |  |  |  |  |  |  |  |  |  |  |  |  |  |  |  |  |
| KY | Native | 7 |  |  |  |  |  |  | 1 |  |  | 2 |  |  | 2 |  |  |  |  |  |  |  |  |  |  |  |  |  |  |  |  |  |  |  |  |
| LYG | Invaded | 6 |  |  |  |  |  |  |  | 6 |  |  |  |  |  |  |  |  |  |  |  |  |  |  |  |  |  |  |  |  |  |  |  |  |  |
| MA | Native | 6 |  |  |  |  |  |  |  | 1 | 3 |  |  |  |  | 1 | 1 |  |  |  |  |  |  |  |  |  |  |  |  |  |  |  |  |  |  |
| MI | Native | 1 |  |  |  |  |  |  |  |  | 10 | 1 |  |  |  |  |  |  |  |  |  |  |  |  |  |  |  |  |  |  |  |  |  |  |  |
| MN | Native | 11 |  |  |  |  |  |  |  |  |  |  |  |  |  |  |  |  |  |  | 1 |  |  |  |  |  |  |  |  |  |  |  |  |  |  |
| NB | Invaded | 13 |  |  |  |  |  |  |  |  |  |  |  |  |  |  |  |  |  |  |  |  |  |  |  |  |  |  |  |  |  |  |  |  |  |
| NC | Invaded | 13 |  |  |  |  |  |  |  |  |  |  |  |  |  |  |  |  |  |  |  |  |  |  |  |  |  |  |  |  |  |  |  |  |  |
| NJ | Invaded | 9 |  |  |  |  |  |  |  | 3 |  |  |  |  |  |  |  |  |  |  |  |  |  |  |  |  |  |  |  |  |  |  |  |  |  |
| NT | Invaded | 12 |  |  |  |  |  |  |  |  |  |  |  |  |  |  |  |  |  |  |  |  |  |  |  |  |  |  |  |  |  |  |  |  |  |
| NX | Invaded | 14 |  |  |  |  |  |  |  |  |  |  |  |  |  |  |  |  |  |  |  |  |  |  |  |  |  |  |  |  |  |  |  |  |  |
| NY | Native | 3 |  |  |  |  |  |  |  |  |  | 1 |  |  |  |  |  |  |  |  |  |  | 6 | 1 |  |  |  |  |  |  |  |  |  |  |  |
| OH | Native | 4 |  |  |  |  |  |  |  |  |  | 5 |  |  | 1 |  |  |  |  |  |  |  |  |  | 1 | 1 |  |  |  |  |  |  |  |  |  |
| ORJ | Native | 7 |  |  |  |  |  |  |  |  |  |  |  |  |  |  |  |  |  |  |  |  |  |  |  |  |  |  | 2 | 1 | 2 |  |  |  |  |
| ORP | Native | 10 |  |  |  |  |  |  |  |  |  | 1 |  |  |  |  |  |  |  |  |  |  | 1 |  |  |  |  |  |  |  |  |  |  |  |  |
| PD | Invaded | 12 |  |  |  |  |  |  |  |  |  |  |  |  |  |  |  |  |  |  |  |  |  |  |  |  |  |  |  |  |  |  |  |  |  |
| SZ | Invaded | 12 |  |  |  |  |  |  |  |  |  |  |  |  |  |  |  |  |  |  |  |  |  |  |  |  |  |  |  |  |  |  | 1 |  |  |
| TNJ | Native | 6 |  |  |  |  |  |  |  |  | 2 | 4 |  |  |  |  |  |  |  |  |  |  |  |  |  |  |  |  |  |  |  |  |  |  |  |
| TZ | Invaded | 5 |  |  |  |  |  |  |  | 4 |  | 1 |  |  |  |  |  |  |  |  |  |  |  |  |  |  |  |  |  |  |  |  |  |  |  |
| VA | Native | 12 |  |  |  |  |  |  |  |  |  |  |  |  |  |  |  |  |  |  |  |  |  |  |  |  |  |  |  |  |  |  |  |  |  |
| WC | Invaded | 10 |  |  |  |  |  |  |  | 2 |  |  |  |  |  |  |  |  |  |  |  |  |  |  |  |  |  |  |  |  |  |  |  |  |  |
| WH | Invaded | 4 |  |  |  |  |  |  |  | 8 |  |  |  |  |  |  |  |  |  |  |  |  |  |  |  |  |  |  |  |  |  |  |  |  |  |
| WI | Native | 6 |  |  |  |  |  |  |  |  | 4 | 1 |  |  |  |  |  |  |  |  |  |  |  |  |  |  |  |  |  |  |  |  |  | 1 |  |
| WY | Invaded | 13 |  |  |  |  |  |  |  |  |  |  |  |  |  |  |  |  |  |  |  |  |  |  |  |  |  |  |  |  |  |  |  |  | 1 |
| WZ | Invaded | 11 |  |  |  |  |  |  |  |  |  | 1 |  |  |  |  |  |  |  |  |  |  |  |  |  |  |  |  |  |  |  |  |  |  |  |
| YN | Invaded | 11 |  |  |  |  |  |  |  |  |  |  |  |  |  |  |  |  |  |  |  |  |  |  |  |  |  |  |  |  |  |  |  |  |  |
| CAN | Native |  |  | 6 | 4 |  |  | 1 | 1 |  |  |  |  |  |  |  |  |  |  |  |  |  |  |  |  |  |  |  |  |  |  |  |  |  |  |
| ORB | Native |  |  | 9 |  |  |  |  |  |  |  |  |  |  |  |  |  |  |  |  |  |  |  |  |  |  | 1 | 1 |  |  |  |  |  |  |  |
| ME | Native |  |  |  |  |  |  |  | 2 |  |  |  |  | 17 |  |  |  | 1 | 3 | 1 |  |  |  |  |  |  |  |  |  |  |  |  |  |  |  |
| NH | Native |  |  |  |  |  |  |  |  |  | 1 |  |  | 9 |  |  |  |  |  |  |  | 2 |  |  |  |  |  |  |  |  |  |  |  |  |  |
| PA | Native |  |  |  |  |  |  |  |  |  |  |  |  |  |  |  |  |  |  |  |  |  |  |  |  |  |  |  | 3 |  |  | 7 |  |  |  |

***Population codes for Table S2:** BB= Bengbu city; BC= British Columbia; CS= Changsha; FZ= Fuzhou; GL= Guilin; GS= Xinyang; HK= Wuhan; HQ= Minhang; HZ= Hangzhou; IA=Iowa; IL=Illinois; IN=Indiana; JDZ= Jingdezhen; JJ= Jiujiang; KY=Kentucky; LYG= Lianyungang; MA= Massachusetts; MI= Michigan; MN= Minnesota; NB= Ningbo; NC= Nanchang; NJ= Nanjing; NT= Nantong; NX= Huzhou; NY= New York; OH=Ohio; ORJ= Oregon 1; ORP=Oregon 2; PD= Pudong; SZ= Suzhou; TNJ= New Jersey; TZ= Taizhou; VA= Virginia; WC= Wuchang; WH= Wuhu; WI= Wisconsin; WY= Wuyi; WZ= Wenzhou; YN= Yunnan; CAN=British Columbia; ORB=Oregon 3; ME=Maine; NH=Rhode Island; PA= Pennsylvanian.

**
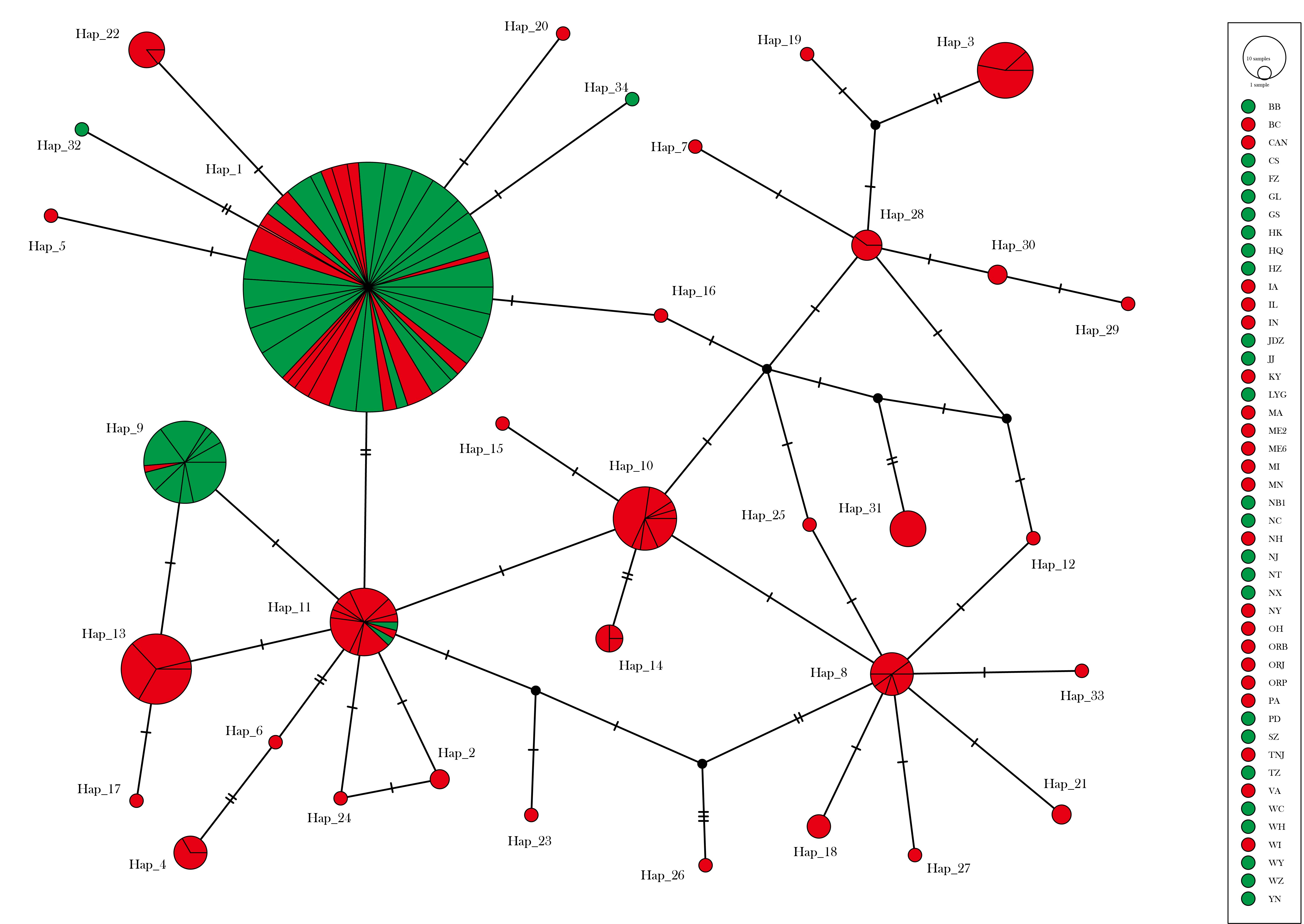
**

**Appendix 7.** A minimum spanning tree showing genealogical relationships among 34 haplotypes (Hap1–Hap34) detected in invasive and native populations of *Solidago canadensis*. Each line connecting the ellipses represents a mutational step between haplotypes. Haplotypes marked in green were found in the invaded range only, haplotypes marked in red occurred exclusively in the native range, while haplotypes marked in both green and red occurred in both ranges. Sizes of haplotypes are proportional to their frequencies. Each sector of a circle represents a distinct population. Black ellipses represent unsampled or missing intermediates.

**Appendix 8.** Hierarchical analysis of molecular variance (AMOVA) based on chloroplast spacer regions *(psbA-trnH and trnL-F)* testing for genetic variation among and within populations of *Solidago canadensis* in the invaded and native ranges. The analyses were based on purely hexaploid populations in the invaded range and mixed-ploidy populations in the native range that contained at least one hexaploid cytotype. The F_ST_ values are global.

| Source of variation | d.f | Sum of square | Variance components | Percentage of variation | *P* |
| --- | --- | --- | --- | --- | --- |
| 1. **Global data set** |  |  |  |  |  |
| Among groups | 1 | 328.65 | 1.16 | 7.76 | **0.000** |
| Among populations within groups | 37 | 2674.98 | 5.32 | 35.65 | **0.000** |
| Within populations | 429 | 3624.38 | 8.45 | 56.59 | **0.0186** |
| Total | 467 | 6628.01 | 14.93 |  |  |
| F_ST_=0.434 |  |  |  |  |  |
| 1. **Invaded range** |  |  |  |  |  |
| Among populations | 23 | 1437.98 | 4.44 | 32.90 | **0.001** |
| Within populations | 265 | 2400.72 | 9.06 | 67.10 | **0.001** |
| Total | 288 | 3838.69 | 13.50 |  |  |
| F_ST_=0.329 |  |  |  |  |  |
| 1. **Native range** |  |  |  |  |  |
| Among populations | 14 | 1237.004 | 6.78 | 47.60 | **0.000** |
| Within populations | 164 | 1223.67 | 7.46 | 52.40 | **0.000** |
| Total | 178 | 2460.67 | 14.24 |  |  |
| F_ST_=0.476 |  |  |  |  |  |


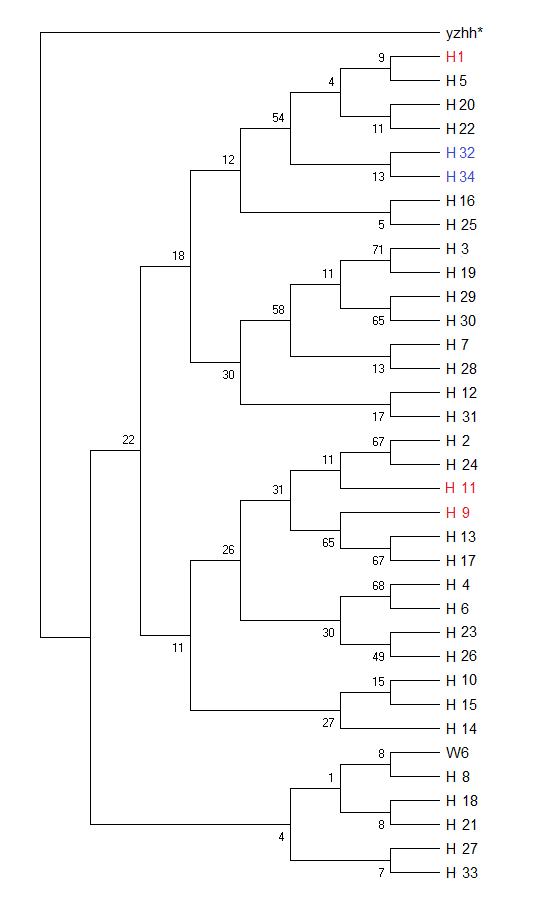


**Appendix 9.** A phylogram of 80 *Solidago canadensis* populations that were sampled in the invaded range in China and Native range in North America. The phylogram was obtained from the Maximum Likelihood analysis of 34 haplotypes based on two chloroplast spacer regions (*psbA-trnH* and *trnL-F*). Numbers on branches are bootstrap support values. Haplotypes marked in red font (H1, H9 and H11) occurred in both invaded and native ranges, haplotypes marked in black font (H2-H8, H10, H12-H31 and H33) occurred exclusively in the native range, while haplotypes in blue font (H32 and H34) occurred exclusively in the invaded range. As outgroups, we used haplotypes W6 and yzhh that were generated, respectively, from DNA of *S. canadensis* seeds obtained from the Germplasm Resources Information Network of the United States Department of Agriculture (accession number W6 52837) and a congener *S. decurrens* that was field-collected in China.


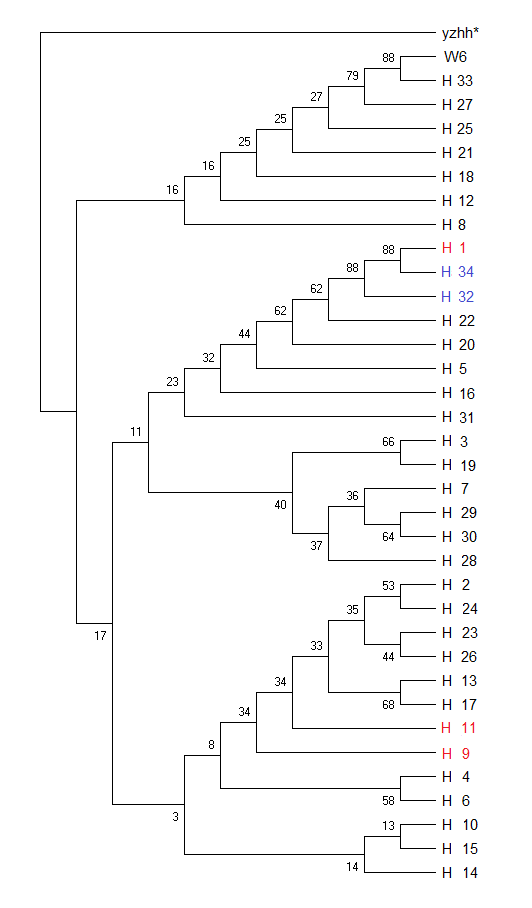


**Appendix 10.** A phylogram of 80 *Solidago canadensis* populations that were sampled in the invaded range in China and Native range in North America. The phylogram was obtained from the Maximum Parsimony analysis of 34 haplotypes based on two chloroplast spacer regions (*psbA-trnH* and *trnL-F*). Numbers on branches are bootstrap support values. Haplotypes marked in red font (H1, H9 and H11) occurred in both invaded and native ranges, haplotypes marked in black font (H2-H8, H10, H12-H31 and H33) occurred exclusively in the native range, while haplotypes in blue font (H32 and H34) occurred exclusively in the invaded range. As outgroups, we used haplotypes W6 and yzhh that were generated, respectively, from DNA of *S. canadensis* seeds obtained from the Germplasm Resources Information Network of the United States Department of Agriculture (accession number W6 52837) and a congener *S. decurrens* that was field-collected in China.

**
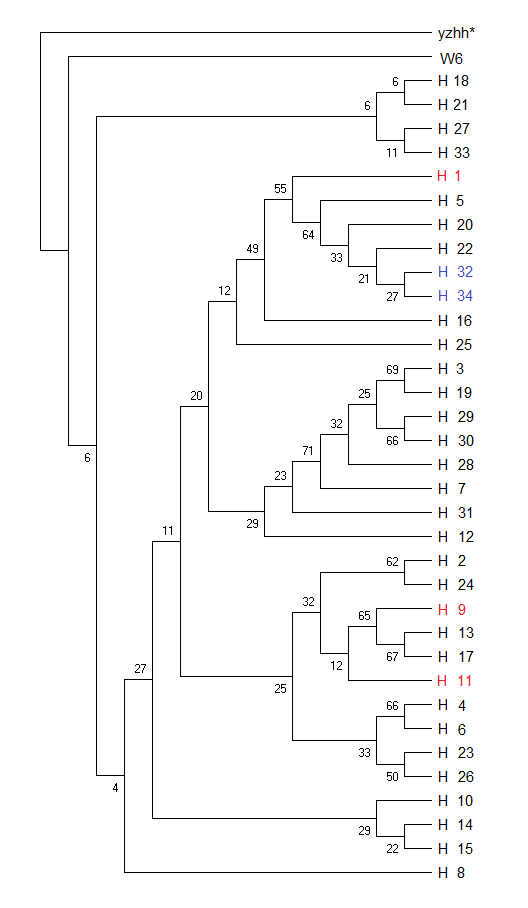
**

**Appendix 11.** A phylogram of 80 *Solidago canadensis* populations that were sampled in the invaded range in China and Native range in North America. The phylogram was obtained from the Neighbor-Joining tree analysis of 34 haplotypes based on two chloroplast spacer regions (*psbA-trnH* and *trnL-F*). Numbers on branches are bootstrap support values. Haplotypes marked in red font (H1, H9 and H11) occurred in both invaded and native ranges, haplotypes marked in black font (H2-H8, H10, H12-H31 and H33) occurred exclusively in the native range, while haplotypes in blue font (H32 and H34) occurred exclusively in the invaded range. As outgroups, we used haplotypes W6 and yzhh that were generated, respectively, from DNA of *S. canadensis* seeds obtained from the Germplasm Resources Information Network of the United States Department of Agriculture (accession number W6 52837) and a congener *S. decurrens* that was field-collected in China.
